# The GENCODE pseudogene resource

**DOI:** 10.1186/gb-2012-13-9-r51

**Published:** 2012-09-05

**Authors:** Baikang Pei, Cristina Sisu, Adam Frankish, Cédric Howald, Lukas Habegger, Xinmeng Jasmine Mu, Rachel Harte, Suganthi Balasubramanian, Andrea Tanzer, Mark Diekhans, Alexandre Reymond, Tim J Hubbard, Jennifer Harrow, Mark B Gerstein

**Affiliations:** 1 Program in Computational Biology and Bioinformatics, Yale University, Bass 432, 266 Whitney Avenue, New Haven, CT 06520, USA; 2 Department of Molecular Biophysics and Biochemistry, Yale University, 266 Whitney Ave, New Haven, CT 06520, USA; 3 Wellcome Trust Sanger Institute, Welcome Trust Campus, Hinxton, Cambridge CB10 1SA, UK; 4 Center for Integrative Genomics, University of Lausanne, Genopode building, Lausanne, 1015, Switzerland; 5 Center for Biomolecular Science and Engineering University of California, 1156 High Street, Santa Cruz, CA 95064, USA; 6 Centre for Genomic Regulation (CRG) and UPF, Dr, Aiguader, 08003 Barcelona, Catalonia, Spain; 7 Department of Computer Science, Yale University, 51 Prospect Street, New Haven, CT 06511, USA

## Abstract

**Background:**

Pseudogenes have long been considered as nonfunctional genomic sequences. However, recent evidence suggests that many of them might have some form of biological activity, and the possibility of functionality has increased interest in their accurate annotation and integration with functional genomics data.

**Results:**

As part of the GENCODE annotation of the human genome, we present the first genome-wide pseudogene assignment for protein-coding genes, based on both large-scale manual annotation and *in silico *pipelines. A key aspect of this coupled approach is that it allows us to identify pseudogenes in an unbiased fashion as well as untangle complex events through manual evaluation. We integrate the pseudogene annotations with the extensive ENCODE functional genomics information. In particular, we determine the expression level, transcription-factor and RNA polymerase II binding, and chromatin marks associated with each pseudogene. Based on their distribution, we develop simple statistical models for each type of activity, which we validate with large-scale RT-PCR-Seq experiments. Finally, we compare our pseudogenes with conservation and variation data from primate alignments and the 1000 Genomes project, producing lists of pseudogenes potentially under selection.

**Conclusions:**

At one extreme, some pseudogenes possess conventional characteristics of functionality; these may represent genes that have recently died. On the other hand, we find interesting patterns of partial activity, which may suggest that dead genes are being resurrected as functioning non-coding RNAs. The activity data of each pseudogene are stored in an associated resource, psiDR, which will be useful for the initial identification of potentially functional pseudogenes.

## Background

Pseudogenes are defined as defunct genomic loci with sequence similarity to functional genes but lacking coding potential due to the presence of disruptive mutations such as frame shifts and premature stop codons [1-4]. The functional paralogs of pseudogenes are often referred to as parent genes. Based on the mechanism of their creation, pseudogenes can be categorized into three large groups: (1) processed pseudogenes, created by retrotransposition of mRNA from functional protein-coding loci back into the genome; (2) duplicated (also referred to as unprocessed) pseudogenes, derived from duplication of functional genes; and (3) unitary pseudogenes, which arise through *in situ *mutations in previously functional protein-coding genes [1,4-6].

Different types of pseudogenes exhibit different genomic features. Duplicated pseudogenes have intron-exon-like genomic structures and may still maintain the upstream regulatory sequences of their parents. In contrast, processed pseudogenes, having lost their introns, contain only exonic sequence and do not retain the upstream regulatory regions. Processed pseudogenes may preserve evidence of their insertion in the form of polyadenine features at their 3' end. These features of processed pseudogenes are shared with other genomic elements commonly known as retrogenes [7]. However, retrogenes differ from pseudogenes in that they have intact coding frames and encode functional proteins [8]. The composition of different types of pseudogenes varies among organisms [9]. In the human genome, processed pseudogenes are the most abundant type due to a burst of retrotranspositional activity [10] in the ancestral primates 40 million years ago [11-13].

Pseudogenes have long been considered as nonfunctional genomic sequences. However, evidence of transcription and conservation of some pseudogenes led to the speculation that they might be functional [14,15], and several estimates of the number of transcribed pseudogenes have been published in recent years [14,16,17]. More recently, studies have shown that, in some cases, expressed pseudogenes can perform crucial regulatory roles through their RNA products [18-21].

Pseudogenes have been suggested to exhibit different types of activity. Firstly, they can regulate the expression of their parent gene by decreasing the mRNA stability of the functional gene through their over-expression. A good example is the *MYLKP1 *pseudogene, which is up-regulated in cancer cells [22]. The transcription of *MYLKP1 *creates a non-coding RNA (ncRNA) that inhibits the mRNA expression of its functional parent, *MYLK*. Moreover, studies in *Drosophila *and mouse have shown that small interfering RNA (siRNA) derived from processed pseudogenes can regulate gene expression by means of the RNA-interference pathway [19,20,23-25], thus acting as endogenous siRNAs. In addition, it has also been hypothesized that pseudogenes with high sequence homology to their parent genes can regulate their expression through the generation of anti-sense transcripts. A recent study by Hawkins and Morris [26] has shown that knocking down a ncRNA antisense to an *Oct4 *pseudogene increases the expression of both *Oct4 *and its pseudogene. Finally, pseudogenes can compete with their parent genes for microRNA (miRNA) binding, thereby modulating the repression of the functional gene by its cognate miRNA. For example, the pseudogene of *PTEN*, a crucial tumor suppressor, regulates the expression of its parent gene following this mechanism [19]. The 3' UTR of the transcript originating from the pseudogene, *PTENP1*, acts as a decoy for the miRNA that represses the parent gene. It has been suggested that this could be a general mechanism of regulation in cancer [27].

While the above examples clearly illustrate that some pseudogenes indeed have a functional role, the extent of this phenomenon is not clear. The large corpus of functional data from the ENCODE consortium provides us with an opportunity to study pseudogene transcription and activity in a systematic and comprehensive manner. It is of interest to study whether these examples are just sporadic exceptions, or indeed represent a generic mechanism for gene regulation.

As a part of the GENCODE project, which aims to annotate all evidence-based human gene features with high accuracy [28,29], we carried out a comprehensive and accurate pseudogene annotation for the entire human genome. We combined automated pipelines and manual curation into a production annotation workflow. This allowed us to precisely annotate pseudogene loci and create a consensus set of pseudogenes.

We identified potential transcribed pseudogenes from locus-specific transcription evidence (that is, EST and mRNA data) and high throughput sequencing data (for example, RNA-Seq) [30]. Candidate transcribed pseudogenes were assessed by large-scale RT-PCR-Seq. The experimental results can serve as a benchmark for computational models of pseudogene transcription. Finally, for each tissue tested, a list of transcribed pseudogenes was obtained. The results indicate that pseudogene transcription is predominantly tissue-specific. Using the functional genomics data from the ENCODE consortium together with the pseudogene annotation, we found that the transcribed pseudogenes tend to associate with a more active chromatin state and maintain more active promoter regions, compared to their non-transcribed counterparts. Both the transcription and regulation of pseudogenes exhibit tissue specificity.

Alongside 'fully active' pseudogenes, we also found evidence for pseudogenes showing partial activity patterns. One hypothesis is that these pseudogenes are the result of genomic elements in the process of either losing or gaining function. Thus, we consider pseudogenes showing partial activity as products of 'dying' genes or undergoing a 'resurrection' process. Two well-known examples of 'dying' and 'resurrected' pseudogenes are *ACYL3 *[31] and *XIST *[32], respectively. Partially active pseudogenes form an interesting group of case studies for the evolution and dynamics of function development. There can be different patterns of pseudogene partial activity. For example, duplicated pseudogenes that arise from 'dying' genes may lack transcriptional evidence, but retain some of the upstream control elements from their parents - for example, active transcription factor binding sites (TFBSs) and various levels of chromatin activity. However, these genomic elements may no longer be evolutionarily constrained. Similarly, we can envision a scenario where processed pseudogenes that do not have their parental upstream regulatory sequences might gain functionality when they are inserted into a region of the genome favorable for transcription. Such pseudogenes may gain upstream regulatory sequences and hence transcriptional potential resulting in novel ncRNAs. The resurrection motif was previously used by Vinckenbosch *et al*. [7] and Kaessmann *et al*. [33] to describe the transition of retrogenes to fully functional genes. The authors suggest that retrogenes 'hitch-hike' on the regulatory apparatus of nearby genes in order to obtain transcription potential.

All the pseudogene activity data generated by this study are recorded in a pseudogene annotation resource file where each pseudogene is 'decorated' with metadata regarding transcription status, functional genomics information, and selection pressure derived from corresponding data. The annotation file is available online [34,35].

## Results

### Assignment of pseudogenes

#### Genome-wide pseudogene identification

**Figure 1:**
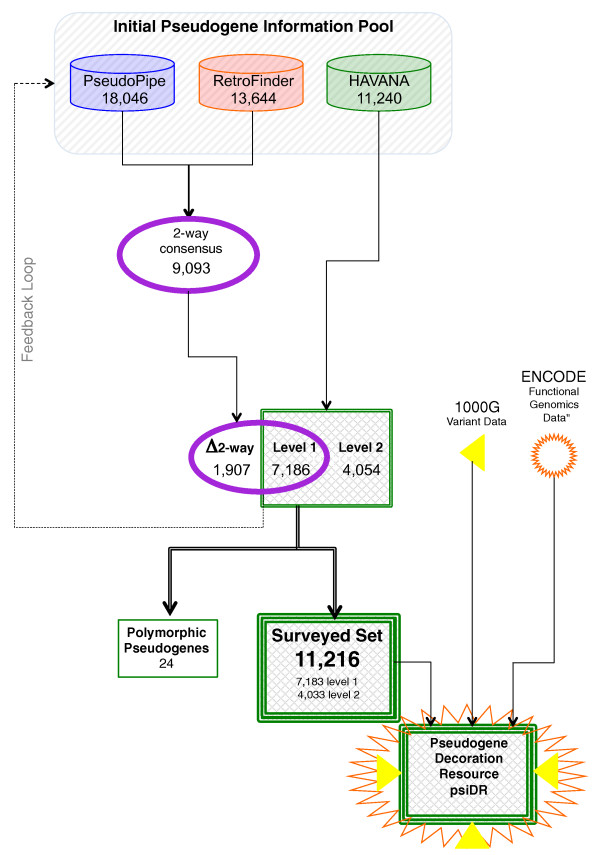
**Pseudogene annotation flowchart**. A flowchart to describe the GENCODE pseudogene annotation procedure and the incorporation of functional genomics data from the 1000 Genomes (1000G) project and ENCODE. This is an integrated procedure including manual annotation done by the HAVANA team and two automated prediction pipelines: PseudoPipe and RetroFinder. The loci that are annotated by both PseudoPipe and RetroFinder are collected in a subset labeled as '2-way consensus', which is further intersected with the manually annotated HAVANA pseudogenes. The intersection results in three subsets of pseudogenes. Level 1 pseudogenes are loci that have been identified by all three methods (PseudoPipe, RetroFinder and HAVANA). Level 2 pseudogenes are loci that have been discovered through manual curation and were not found by either automated pipeline. Delta 2-way contains pseudogenes that have been identified only by computational pipelines and were not validated by manual annotation. As a quality control exercise to determine completeness of pseudogene annotation in chromosomes that have been manually annotated, 2-way consensus pseudogenes are analyzed by the HAVANA team to establish their validity and are included in the manually annotated pseudogene set if appropriate. The final set of pseudogenes is compared with functional genomics data from ENCODE and genomic variation data from the 1000 Genomes project.

The annotation of all pseudogenes in the human reference genome is part of the wider effort by the GENCODE consortium that also aims to identify all protein-coding, long non-coding RNA (lncRNA) and short RNA genes [28,29]. Similar to the annotation of other functional classes, the annotation of pseudogenes contains models that have been created by the Human and Vertebrate Analysis and Annotation (HAVANA) team, an expert manual annotation team at the Wellcome Trust Sanger Institute. This is informed by, and checked against, computational pseudogene predictions by the PseudoPipe [36] and RetroFinder [37] pipelines (details in Materials and methods). These computational pseudogene predictions provide hints to manual annotators during the first-pass of annotation and identify potential missing features, flagging them for manual re-investigation (Figure 1).

A 'level' index is used to represent the supportive evidence of each pseudogene from the annotation procedure. Level 1 indicates pseudogenes that have been confirmed by both manual and automatic annotation pipelines. Level 2 highlights elements that have been annotated by manual inspection only. We also define level A as pseudogenes determined by automated annotation. This is represented as PseudoPipe-specific (A-P), RetroFinder-specific (A-R) and a '2-way' consensus set derived from predictions of both pipelines (2-way). Table 1 gives a summary of the pseudogenes used in GENCODE v7 based on their annotation level.

**Table 1:** Pseudogenes used in GENCODE v7

Level	Pseudogenes
Level 1^a,b^	7,186
Level 2^a,b^	4,054
Polymorphic^b^	24
Level A-P	18,046
Level A-R	13,644
Havana^a^	11,240
'2-way' consensus^a^	9,093
Δ-'2-way' consensus	1,907

^a^Including polymorphic pseudogenes. ^b^Excluding ENSEMBL annotated pseudogenes.

The pseudogenes are annotated with different biotypes (for example, processed or duplicated) based on the mechanism by which they arose and their evolutionary histories. The pseudogene biotypes are explained in detail in Table 2.

**Table 2:** Pseudogene biotypes

Biotype	Definition
Processed pseudogene	Pseudogene created via retrotransposition of the mRNA of a functional protein-coding parent gene followed by accumulation of disabling mutations
Duplicated pseudogene	Pseudogene created via genomic duplication of a functional protein-coding parent gene followed by accumulation of disabling mutations
Unitary pseudogene	Pseudogene for which the ortholog in a reference species (mouse) is coding but the human locus has accumulated fixed disabling mutations
Polymorphic pseudogene	Locus known to be coding in some individuals but with disabling mutations in the reference genome
IG pseudogene	Immunoglobulin gene segment with disabling mutations
TR pseudogene	T-cell receptor gene segment with disabling mutations

The GENCODE protein-coding and pseudogene annotation is completely integrated. Each potential pseudogene locus is investigated for protein-coding potential (and vice versa) and all loci are strictly described as either protein-coding or pseudogenic, but never both (Figure S0 in Additional file 1). Protein-coding loci derived via retrotransposition may be misidentified as processed pseudogenes due to the structural differences when compared to their parent loci (reviewed by Kaessmann *et al*. [33]). However, we distinguish retrogenes from processed pseudogenes by careful manual annotation (Table S0 in Additional file 1). For example, the retrotransposed protein-coding loci *USP26*, *KLF14 *and *PGK2 *are all protein-coding biotypes in the GENCODE geneset.

In this study, we focused on a pseudogene set composed of manually annotated pseudogenes (a union of levels 1 and 2). Polymorphic pseudogenes, which are coding genes that are pseudogenic due to the presence of a polymorphic premature stop codon in the reference genome (GRCh37), were excluded from our study in order to avoid the likelihood that they may have coding potential in the cell lines and tissues studied by other ENCODE groups. We call these 11,216 pseudogenes the 'surveyed set'. The set contains 138 unitary pseudogenes. For the purpose of this paper, only the processed and duplicated pseudogenes will be discussed in detail.

The workflow used to identify the pseudogenes in this dataset is described in Figure 1. In addition to the 11,216 pseudogenes, the '2-way' consensus set derived from the automated pipeline annotations includes an additional 1,910 pseudogenes (including 3 level 1 polymorphic pseudogenes). As manual annotation is done in a chromosome-by-chromosome fashion, it is not biased relative to any particular genomic feature. Thus, we feel that our 'surveyed set' is the best representative of the total pseudogene complement in the genome.

#### Pseudogene statistics

**Figure 2:**
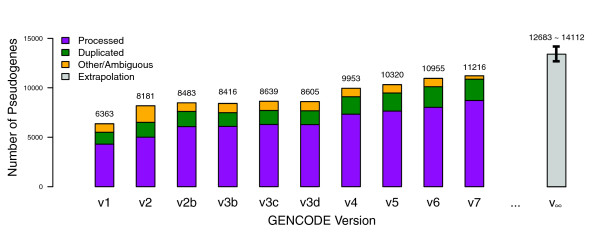
**Growth of pseudogene annotation**. The numbers of pseudogenes present in the GENCODE dataset from version 1 to version 7 are plotted. The three colors - purple, green and yellow - represent processed, duplicated and other types of pseudogenes, respectively. The pseudogenes were annotated manually and/or using the automated pipelines PseudoPipe and RetroFinder. The gray bar indicates the estimated number of pseudogenes (± standard deviation present in the human genome.

The number of manually annotated pseudogenes in the human genome has grown along with the development of the GENCODE project. Figure 2 follows the variation of the total number of pseudogenes in the human genome with the development of GENCODE annotation from v1 to v7. Over all the GENCODE releases, the total number of pseudogenes follows a linear growth rate. Extrapolating from this tendency, we estimate that the entire human genome contains approximately 12,683 protein pseudogenes. Alternatively, using the current manually annotated pseudogenes as a benchmark, we can estimate the accuracy of the automated pipelines, and then extrapolate it to the whole genome. With this approach, we estimated that the number of pseudogenes in the human genome is 14,112 (Figure 2). Details of both approaches are described in Materials and methods.

The estimated number of pseudogenes in this study is smaller than that predicted from the pilot study, where we identified 201 pseudogenes in 1% of the human genome. One reason is that the pilot study included biased genomic regions - there was a single region containing a large cluster of olfactory receptor pseudogenes - and is not representative of the entire human genome [16]. These estimates are smaller than previous computational analyses reported by Torrents *et al*. [11] and Zhang *et al*. [38] that predicted the presence of 19,724 and 19,293 pseudogenes, respectively. This is due to improvement in the genome assembly and the gene annotation datasets. The number of genes annotated in the genome has steadily dropped with the improvement in annotation [39]. Consequently, the total number of pseudogenes decreased due to a smaller and more accurate number of parent proteins. Thus, spurious pseudogene annotations due to erroneous gene models are no longer present in the current pseudogene dataset.

#### Difficulties in pseudogene annotation

The hybrid approach of pseudogene identification combining manual and automated annotation allows us to take advantage of the strengths of both methods. Automated pipelines for the detection of pseudogenes have significant strengths, such as fast speed, comprehensive coverage and ability to detect weak homologies revealing highly degraded or truncated pseudogenes. In addition, the pipelines can be combined with comparative analysis to highlight the evolutionary origin of pseudogenes (for example, to determine whether a single exon pseudogene has arisen due to duplication or a *de novo *retrotransposition event). However, automated methods are likely to introduce or propagate errors due to either mis-annotation of parent loci or lack of a genome-wide high-quality annotation of protein-coding genes. The latter fact probably accounts for the large number of pseudogenes in the initial pipeline surveys.

One difficult case for pseudogene annotation is the identification of partially spliced pseudogenes, derived via the retrotransposition of a transcript that retains at least one intron for the parent locus. We have identified a total of eight such partially processed pseudogenes through computational analysis followed by careful manual examination (Table S3 in Additional file 1).

Manual intervention allows the assessment of the validity of a protein-coding locus used as a parent by an automated pseudogene prediction method. It is also essential in both identifying and elucidating those instances where pseudogenes intersect with other transcript biotypes, that is, protein-coding loci and lncRNAs, such as in the case of resurrected pseudogenes. These pseudogenes often require only relatively small changes in structure, like a single exon skip or shifted splice junction, to restore coding potential and thus are challenging to detect computationally. Several cases where pseudogenes intersect with functional loci are discussed below.

#### Pseudogene sequences used by other functional loci

**Figure 3:**
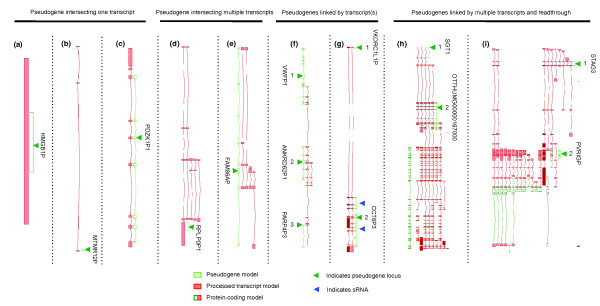
**Complexity of transcribed pseudogenes**. Screenshots of pseudogene annotation are taken from the Zmap annotation interface. The pseudogenes are represented as open green boxes and indicated by dark green arrowheads, exons of associated transcript models are represented as filled red boxes and connections are shown by red lines. The coding exons of protein-coding models are represented by dark green boxes and UTR exons as filled red boxes; protein-coding models are also indicated by red arrowheads. **(a-c) **Single pseudogene models intersecting with single transcript models. (a) The processed pseudogene High mobility group box 1 pseudogene (*HMGB1P*; HAVANA gene ID: OTTHUMG00000172132 and its associated unspliced (that is, single exon) transcript. (b) The processed pseudogene Myotubularin related protein 12 pseudogene (*MTMR12P*; HAVANA gene ID: OTTHUMG00000167532) and a spliced transcript model with three exons. (c) A duplicated pseudogene PDZ domain containing 1 pseudogene 1 (*PDZK1P1*; HAVANA gene ID: OTTHUMG00000013746) and a spliced transcript model with nine exons. **(d,e) **Single pseudogene models intersecting with multiple transcripts. (d) The processed pseudogene Ribosomal protein, large, P0 pseudogene 1 (*RPLP0P1*; HAVANA gene ID: OTTHUMG00000158396) and five spliced transcripts. (e) The duplicated pseudogene Family with sequence similarity 86, member A pseudogene (*FAM86AP*; HAVANA gene ID: OTTHUMG00000159782) and four spliced transcripts. **(f,g) **Groups of multiple pseudogenes that are connected by overlapping transcripts. (f) Three pseudogenes with single connecting transcripts: 1 is the duplicated pseudogene von Willebrand factor pseudogene 1 (*VWFP1*; HAVANA gene ID: OTTHUMG00000143725); 2 is a duplicated pseudogene ankyrin repeat domain 62 pseudogene 1 (*ANKRD62P1*; HAVANA gene ID: OTTHUMG00000149993); 3 is the duplicated pseudogene poly (ADP-ribose) polymerase family, member 4 pseudogene 3 (*PARP4P3*; HAVANA gene ID: OTTHUMG00000142831). Pseudogene 1 and 2 are connected by a seven exon transcript, pseudogenes 2 and 3 are connected by a nine exon transcript and there is a third transcript that shares two of its four exons with pseudogene 2. (g) Two pseudogenes with multiple connecting transcripts: 1 is the processed pseudogene vitamin K epoxide reductase complex, subunit 1-like 1 pseudogene (*VKORC1L1P*; HAVANA gene ID: OTTHUMG00000156633); 2 is the duplicated pseudogene chaperonin containing TCP1, subunit 6 (zeta) pseudogene 3 (*CCT6P3*; HAVANA gene ID: OTTHUMG00000156630). The two pseudogenes are connected by two transcripts that initiate at the upstream pseudogene and utilize a splice donor site within the single exon, which is also a splice donor site in the pseudogene's parent locus. Interestingly, the downstream locus hosts two small nucleolar RNAs (snoRNAs) that are present in the parent locus and another paralog. **(h) **A very complex case where multiple pseudogenes, connected by multiple transcripts, read through into an adjacent protein-coding locus: 1 is the duplicated pseudogene suppressor of G2 allele of SKP1 (*S. cerevisiae*) pseudogene (*SGT1P*; HAVANA gene ID: OTTHUMG00000020323); 2 is a novel duplicated pseudogene (OTTHUMG00000167000); and the protein-coding gene is *C9orf174*, chromosome 9 open reading frame 174 (OTTHUMG00000167001). **(i) **A similarly complex case where multiple pseudogenes, connected by multiple transcripts, read through into an adjacent protein-coding locus: 1 is a duplicated pseudogene stromal antigen 3 pseudogene (*STAGP3*; HAVANA gene ID: OTTHUMG00000156884); 2 is a duplicated pseudogene poliovirus receptor related immunoglobulin domain containing pseudogene (*PVRIGP*; HAVANA gene ID: OTTHUMG00000156886); and the protein-coding gene is *PILRB*, paired immunoglobin-like type 2 receptor beta (OTTHUMG00000155363). sRNA, small RNA.

Pseudogenes can contribute sequences to other loci, including coding exons, 5' UTR, 3' UTR and polyadenine signals, via their insertion in either the sense or antisense orientation. Such loci range in complexity from simple cases where a single pseudogene is overlapped by one transcript to instances of greater complexity where multiple pseudogenes are overlapped by multiple transcripts, and transcriptional read-through from proximal protein-coding and lncRNA loci (Figure 3). For example, *MST1P9 *(Figure S1 in Additional file 1), whose translation is supported by mass spectrometry data, is a potential 'resurrected' pseudogene that has gained a novel function and therefore has been re-annotated as a new protein-coding locus [29]. Another example is the *PTEN *pseudogene [19], which has been resurrected as a functioning lncRNA that regulates its parent locus via an intermediate pathway involving shared miRNAs. In all these cases, good annotation highlights the evolutionary history of pseudogene-derived loci, which may give insight into any potentially new function.

We manually examined 131 pseudogene models overlapping protein-coding genes. Within this set, 80 pseudogenes are annotated on the same strand as the protein-coding gene, of which 52 are duplicated and 28 are processed pseudogenes. Pseudogenes overlapping annotations on different strands comprise 20 duplicated and 31 processed pseudogenes. All the pseudogenes overlapping protein-coding genes fell into one of the following categories (Figures S2 and S3 in Additional file 1): (1) part of the pseudogene sequence is used to create a new alternatively spliced internal exon in the protein-coding gene (Figure S2a in Additional file 1); (2) the pseudogene sequence contributes the 5' terminal exon of the protein-coding gene (Figure S2b in Additional file 1); (3) the pseudogene sequence contributes the 3' terminal exon of the protein-coding gene (Figure S2c in Additional file 1).

The role of processed pseudogenes in the evolution of protein-coding genes has already been described [37]. Here we have found the same to be true for duplicated pseudogenes. Further analysis is required to determine whether the translation of the acquired exon is in the same or different frame to the coding sequence of the pseudogene's parent and to determine whether splice sites are shared between the overlapping genes.

### Pseudogene Decoration Resource (psiDR)

There is a large amount of information related to pseudogene annotation that goes considerably beyond simple genomic coordinates. To facilitate the study of pseudogene activity, we have created a resource to 'decorate' the pseudogene annotation with additional information - the Pseudogene Decoration Resource (psiDR). To create this resource, we consistently collected and organized a large variety of genomic information relating to each pseudogene in a consistent manner, such as transcriptional activity, chromatin features, functional genomics and evolutionary constraint. As described in the following sections, various models and filters were applied to the corresponding data to characterize biological features of pseudogenes. We characterized the transcriptional state of pseudogenes using the integration of three pipelines. Furthermore, we used simple statistical models to partition the pseudogenes based on various genomic features. The distribution of functional genomics and selection signals was compared between transcribed and non-transcribed pseudogenes. Finally, quantifiers were assigned to each pseudogene according to the output of the model, such as whether it has an active chromatin state, associates with active promoter regions, and so on. Tissue/cell line-specific information was recorded wherever applicable.

Overall, psiDR provides a variety of activity information for all the surveyed pseudogenes. It is a valuable resource for pseudogene activity studies that can provide potential targets for further experimental follow-up. Table 3 contains a detailed description of the pseudogene information featured in psiDR. In the following sections, we describe each component in detail.

**Table 3:** Fields for pseudogene features in the psiDR annotation file

Field	Explanation	psiDR value
Transcript ID	Pseudogene ID from GENCODE annotation. Used for cross-referencing	
Parent	Protein ID, Gene ID, chromosome, start, end and strand. Detailed in section *'Parents of pseudogenes'*	
Sequence similarity	The percentage of pseudogene sequence preserved from parent	
Transcription	Evidence for pseudogene transcription and validation results. May be tagged as EST, BodyMap, RT-PCR or None, which represent pseudogene expression evidence from corresponding data sources. Multiple tags are separated by commas. Detailed in section *'Transcription of pseudogenes'*	1, transcription; 0, otherwise
DNaseI hypersensitivity	A categorical result indicating whether the pseudogene has easily accessible chromatin, predicted by a model integrating DNaseI hypersensitivity values within 4 kb genomic regions upstream and downstream of the 5' end of pseudogenes. Detailed in section *'Chromatin signatures of pseudogenes'*	1, has Dnase hypersensitivity in upstream; 0, otherwise
Chromatin state	Whether a pseudogene maintains an active chromatin state, as predicted by a model using Segway segmentation. Detailed in section *'Chromatin signatures of pseudogenes'*	1, active chromatin; 0, otherwise
Active Pol2* binding	Whether Pol2 binds to the upstream region of a pseudegene. Detailed in section *'Upstream regulatory elements'*	1, active binding site; 0, otherwise
Active promoter region	Whether there are active promoter regions in the upstream of pseudogenes. Detailed in section *'Upstream regulatory elements'*	1, active binding site; 0, otherwise
Conservation	Conservation of pseudogenes is derived from the divergence between human, chimp and mouse DNA sequences. Detailed in section *'Evolutionary constraint on pseudogenes'*	1, conserved; 0, otherwise

*Pol2, RNA polymerase II.

### Parents of pseudogenes

#### Identification of pseudogene parents

We refer to the functional paralog with the greatest sequence similarity to a pseudogene as its parent gene. Identifying pseudogene parents is critical for the study of a pseudogene's evolutionary history and its potential regulatory functions. Currently, we have successfully identified parents for 9,368 pseudogenes, whereas the parents for the remaining 1,848 pseudogenes are still ambiguous and may require further manual annotation. It is important to note, however, that it is not always possible to identify the true parent of a pseudogene with certainty. For example, when a pseudogene is highly degraded and is derived from a parent gene with highly similar paralogs, or when the parent contains a commonly found functional domain.

The total number of parent genes for all the pseudogenes is 3,391. While most parents (2,071) have just one pseudogene, some of them are associated with a large number of pseudogenes, among which are ribosomal protein L21 (*RPL21*; 143 pseudogenes) and glyceraldehyde-3-phosphate dehydrogenase (*GAPDH*; 68 pseudogenes). These results are consistent with previous studies showing that housekeeping genes tend to have more pseudogenes [13,40,41].

#### Sequence identity to parent genes

Recent studies have shown that some pseudogenes can regulate their parent genes' activity at the transcript level [19,20,23-25]. For example, the pseudogene transcript sequence homologous to the parent may either hybridize with the parent mRNA to generate endogenous siRNAs or act as a decoy to buffer the binding of a miRNA to parent gene transcripts. Pseudogenes with such functionalities are expected to exhibit high sequence identity to their parent genes' coding exons and/or 3' UTR sequences. Therefore, for each pseudogene, it is of interest to examine the sequence identity to its parent in these particular regions.

**Figure 4:**
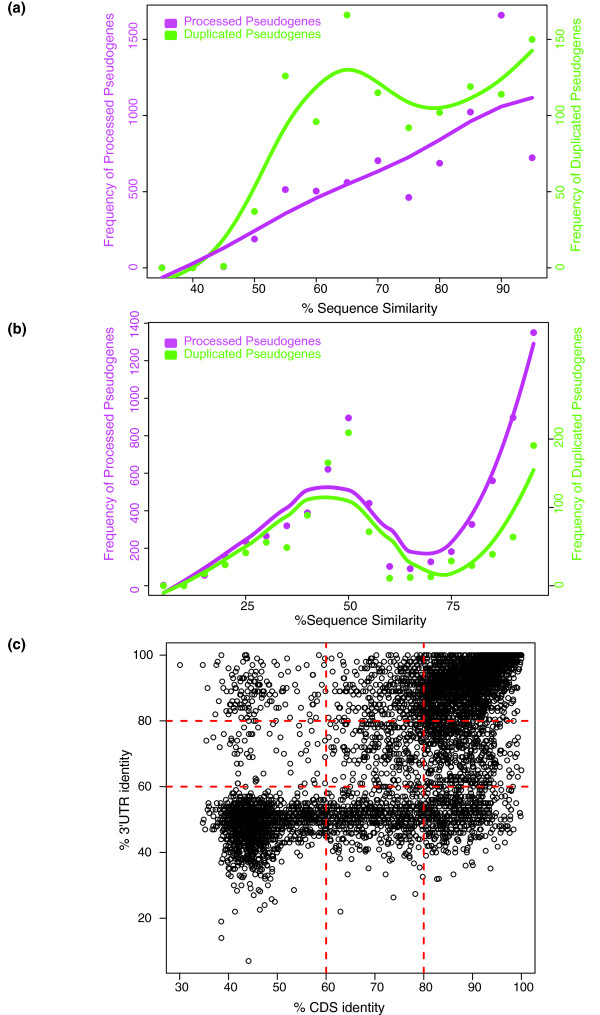
**Sequence identity between pseudogenes and their parents**. **(a) **Distribution of pseudogene sequence identity to coding exons (CDS) of parent genes. **(b) **Distribution of pseudogene sequence identity to 3' UTR of parent genes. **(c) **Scatter plot of sequence identity of all the pseudogenes to the CDS and UTR regions of their parents.

We calculated sequence identity between pseudogenes and their parents by examining the alignment of their exon sequences (see Materials and methods). Processed and duplicated pseudogenes were shown, on average, to have similar sequence identity to their parents' coding sequences (CDSs), with mean identities of 80.3% (±13.2%) and 76.9% (±13.9%), respectively. However, the two classes of pseudogenes exhibit different sequence identity distribution patterns. Processed pseudogenes have a unimodal distribution, with a specific group showing high sequence identity to their parents (around 90%). Duplicated pseudogenes, in contrast, show a more uniform distribution of sequence identities to their parents' CDSs (Figure 4a). These results are in accordance with previous data showing a burst of retrotransposition events in the recent evolutionary history of the human genome that generated a large number of young processed pseudogenes [13,42,43]. The relatively higher number of duplicated pseudogenes with low sequence identity (approximately 65%) to their parents can be an indication of a minor burst in the creation of duplicated pseudogenes in ancient time. Both duplicated and processed pseudogenes show no significant difference in sequence identity to the 3' UTR of their parent genes. The mean sequence identity is 68.4% (±24.9%) for processed pseudogenes and 61.0% (±24.2%) for duplicated pseudogenes. Both processed and duplicated pseudogenes exhibit a bimodal distribution for the 3' UTR sequence identity (Figure 4b), implying that the CDS and 3' UTR of pseudogenes may be under different evolutionary constraints.

We next compared the CDS and 3' UTR sequence identity of each pseudogene to its parent. While most pseudogenes have comparable sequence identities to the two genomic regions, there are pseudogenes that exhibit high sequence identity to the 3' UTR but poor identity to CDS, or vice versa (Figure 4c). This inconsistency implies that mutations were rejected by natural selection non-randomly. Certain regions in the sequence may be under higher evolutionary constraint than the others. We identified 998 pseudogenes showing a high (>80%) sequence identity to parent CDS and simultaneously poor (<60%) sequence identity to the 3' UTR, and 36 pseudogenes with high (>80%) sequence identity to the parent 3' UTR and small (<60%) sequence identity to CDS. These thresholds were selected to separate the two modes of the sequence identity distributions (Figure 4a,b). Using this simple approach, we partitioned the pseudogenes into nine groups based on sequence identity between the pseudogenes and the parent genes at CDS and 3' UTR levels. Each pseudogene has a label corresponding to one of the nine classes, which is recorded in psiDR.

### Transcription of pseudogenes

**Figure 5:**
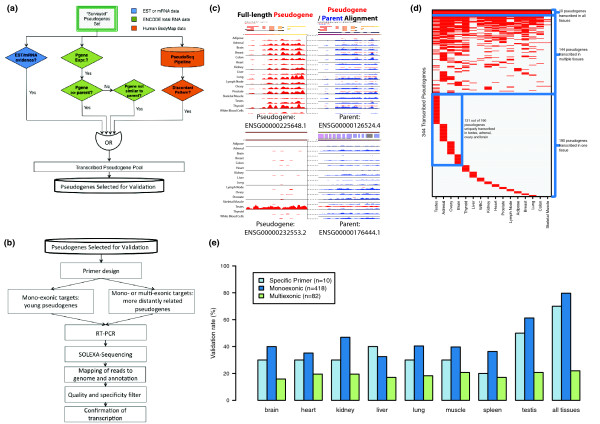
**Transcription of pseudogenes**. **(a) **Pipeline for computational identification of transcribed pseudogenes (Pgenes). The 'OR' gate (binary operator) indicates the acceptance criteria for a candidate to enter the transcribed pseudogene pool. Expressed pseudogene candidates showing transcription evidence in ESTs/mRNAs, total RNA-Seq data, and BodyMap data were sent for wet-lab validation by RT-PCR or RT-PCR-Seq. **(b) **Process flow of experimental evaluation of pseudogene transcription. **(c) **User interface of PseudoSeq for identifying transcribed pseudogenes with BodyMap data. **(d) **Transcribed pseudogenes identified using Human BodyMap data. **(e) **Experimental validation results showing the transcription of pseudogenes in different tissues.

We identified pseudogene transcription on a genome-wide scale by combining computational pipelines (Figure 5a) and high-throughput wet-lab experiments. Transcribed pseudogenes were identified with computational models, from which a selected group was then evaluated experimentally via RT-PCR-Seq techniques (Figure 5b).

#### Pseudogene transcription identified by a sequence of computational pipelines

Three computational pipelines were combined to identify transcribed pseudogenes using various data sources; a pseudogene was considered transcribed and its status was recorded in psiDR if it passed the selection criteria of at least one of the three (Figure 5a). Thus, 876 transcribed pseudogenes were identified that include 531 processed and 345 duplicated ones. We consider this to be a conservative estimate of the total number of transcribed pseudogenes, since each of the pipelines had fairly stringent selection parameters. The three pipelines are described as follows.

The first pipeline examined manually annotated pseudogenes with locus-specific transcription evidence derived from databases of ESTs and mRNAs [30]. The locus-specific transcription evidence consists of a best-in-genome alignment in the pseudogene locus and clear differences when compared to the parent locus. Using this approach, 422 pseudogenes were classified as transcribed.

The second pipeline focused on the total RNA-Seq data, which is available for only two ENCODE cell lines: GM12878 and K562. One advantage of using a total RNA sample lies in its comprehensive inclusion of transcription products such as both mRNAs and small RNAs. In this method, we considered a pseudogene as transcribed if one of the following two criteria was fulfilled: (1) there were reads mapped to the pseudogene sequence and no reads mapped to the parent; or (2) both the pseudogene and the parent were covered by reads but they had a low sequence similarity (<90%). Using this conservative approach, we identified 110 transcribed pseudogenes.

The third pipeline was targeted at pseudogenes showing some transcriptional evidence but not fulfilling the requirements of the second selection pipeline. In this approach we used the PseudoSeq pipeline to analyze the data from the Illumina Human BodyMap 2.0 project. PseudoSeq analyzed the expression patterns of a pseudogene and its parent gene using RNA-Seq data across multiple tissues (Figure 5c). Pseudogenes with discordant expression patterns from those of the parent genes were considered as transcribed. The potential of a mapping artifact was ruled out by the difference in their expression patterns. Using this approach, we identified 344 pseudogenes with transcription evidence (Figure 5d).

#### Experimental validation

We have experimentally tested the transcription evidence of 469 transcribed pseudogenes predicted by computational approaches (see Materials and methods). We used RT-PCR-Seq, a method that combines RT-PCR amplification with a highly multiplexed sequencing readout, that reaches sensitivities of 92% and 79% for known coding and non-coding transcripts, respectively [44].

Targeted pseudogenes can be divided into three classes: (1) multiexonic models in which we assessed an exon-exon junction between exons less than 90% identical to the parent (and other duplicated pseudogene copies); (2) monoexonic models where pseudogene-specific primers could be designed (that is, primers are unable to amplify the parent gene because they map to regions possessing a large number of substitutions between parent and pseudogene); and (3) monoexonic models, where it was not feasible to design specific primers. Therefore, the resulting amplification of both parent and pseudogene transcripts must be discriminated by substitutions present in the amplicon. As monoexonic models are sensitive to genomic DNA contamination, they were assessed by amplification of cDNA in which a dNTP analog was incorporated as described in [45]. Each of these three categories was considered experimentally validated using different criteria (see Materials and methods) [44]. The criteria were adjusted to take advantage of the pseudogene-specific substitutions, as well as to consider the possibility that sequencing reads mapping to the pseudogenes could result from co-amplified expressed parental genes. We validated 7 out of 10 monoexonic pseudogenes targeted with specific primers, and 333 out of 418 regular monoexonic pseudogenes (Figure 5e). The validation did not reach 100%, probably due to fact that some pseudogenes were not being transcribed in the eight tissues tested.

Among the 82 multiexonic pseudogenes, only 18 were experimentally confirmed (41 pseudogenes were also tested with the monoexonic model). This lower validation rate is explained by the fact that the transcribed pseudogenes probably function as lncRNAs rather than being translated into proteins. Thus, it is probable that multiexon pseudogenes will not be spliced in identical fashion to their parent proteins. This is consistent with the results that among the 41 pseudogenes that were tested by both the multiexonic model and the monoexonic model, 4 were validated by both models, 35 were validated by the monoexonic model only, and 2 were not validated by either model.

The testis transcriptome showed the highest complexity (highest percentage of validated expressed pseudogene models at 64% from all three classes combined), which is consistent with the high level of transcription reported in this tissue [44,46]. The expression patterns determined by RT-PCR-Seq are highly correlated with the expression reported by RNA-Seq. For example, the expression patterns of all the monoexonic pseudogenes, validated with specific primers, are fully replicated by RT-PCR-Seq.

### Evolutionary constraint on pseudogenes

Beyond transcription, we next focused on the evolutionary constraint of human pseudogenes. Constraint on genomic sequences has also been regarded as an indicator of biological function [15]. The availability of whole genome sequencing data and personal genome sequencing data allowed us to carry out an evolutionary constraint study on human pseudogenes at a genome-wide scale from both divergence and diversity perspectives.

**Figure 6:**
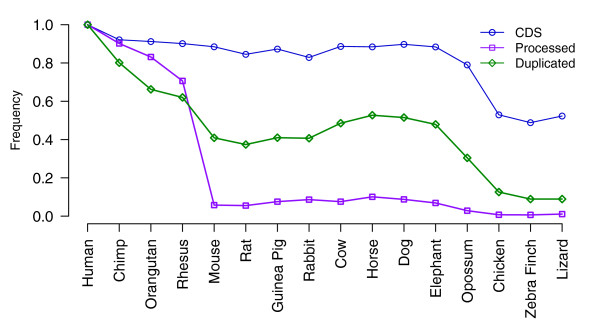
**Preservation of human coding sequences, processed pseudogenes and duplicated pseudogenes**. Sequences orthologous to human genomic regions from different species were studied. The sequence preservation rate was calculated as the percentage of sequences aligned to human sequence from each species. The calculation was based on a MultiZ multiple genome sequence alignment.

Firstly, we analyzed the sequence preservation between human pseudogenes and their orthologs in 15 different organisms ranging from chimpanzee to lizard, where the orthologs were derived from the multiple sequence alignments available from the University of California at Santa Cruz (UCSC) genome browser. Figure 6 shows for each species the preservation rates of protein-coding sequences, duplicated pseudogenes and processed pseudogenes. While the preservation of duplicated pseudogenes decreases gradually with the increase of evolutionary distance of the species from human, the preservation of processed pseudogenes exhibits an abrupt decrease from macaque to mouse and remains low within the species more divergent than mouse. These results are in agreement with previous findings showing that most processed pseudogenes in humans and mice are lineage-specific, arising from distinct retrotransposition bursts happening in the two organisms after they diverged [13,41].

**Figure 7:**
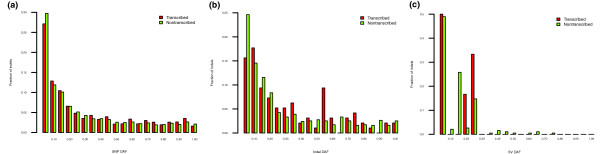
**(a) SNP-, (b) indel-, and (c) SV-derived allele frequency spectra are shown for transcribed and non-transcribed pseudogenes**. The distributions of variant DAFs in transcribed and non-transcribed pseudogenes are not statistically different.

Secondly, we studied the evolutionary selection on human pseudogenes by integrating the annotation with the variation data from the 1000 Genomes pilot project [47]. We computed the densities of SNPs, indels and structural variations in pseudogene sequences and their respective derived allele frequencies. The densities suggested a weak signal for differential selection on transcribed versus non-transcribed pseudogenes (Figure S6 in Additional file 1). However, no significant differences were found in the derived allele frequency spectra (DAF) (Figure 7), and it is possible that the difference in the densities may be due to confounding factors such as variation in mutation rates in the genome. Thus, we cannot make a strong statement about selection in the human population on transcribed pseudogenes.

Next we analyzed the pseudogenes' divergence using sequence identity to orthologs in the chimpanzee genome, where higher sequence identity implies lower divergence and negative selection. The distribution of pseudogenes' divergence was calculated and the results indicate that a fraction of the pseudogenes exhibiting lower divergence are under evolutionary constraint (Figure S5 in Additional file 1).

Divergence and diversity results indicate that although pseudogenes, as a group, are under low selection pressure, a small subset may exhibit higher evolutionary constraint. To identify these pseudogenes, we analyzed the divergence to orthologs in the chimp and the mouse genome under the assumption that the conserved pseudogenes will show significantly lower divergence than neutral background (see Materials and methods). There are 1,019 conserved pseudogenes identified in the human genome. The conserved group is enriched with transcribed pseudogenes (195 conserved pseudogenes are transcribed, *P*-value = 1.19 × 10^-35^), strongly implying biological function. Duplicated and processed pseudogenes are differentially conserved; 28.1% of duplicated pseudogenes and 3.4% of processed pseudogenes are conserved. This difference is due to the fact that most processed pseudogenes are lineage-specific, and also that most of them are dead on arrival. Evolutionary constraint information of all the pseudogenes is collected in the psiDR.

### Chromatin signatures of pseudogenes

Following the study of the canonical signatures of transcription and selection of pseudogenes, we focused on the more elusive indications of 'partial activity' - chromatin marks and upstream transcription factor binding. In particular, we intersected the annotated pseudogene locations in the human genome with the extensive amount of functional genomics data from the ENCODE production project. We were able to correlate these results with the transcription and conservation information of pseudogenes discussed previously, to identify pseudogene cases consistent with partial activity.

In this section, we present the results pertaining to chromatin state. Chromatin accessibility, histone modification and genome-wide segmentation pattern on ENCODE cell lines were studied and results for the K562 cell line are described and shown here as an example.

#### Chromatin accessibility and histone marks of pseudogenes

**Figure 8:**
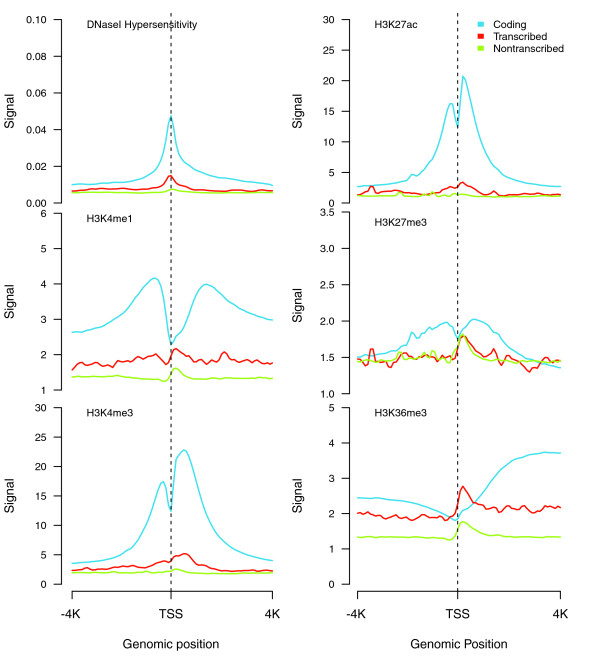
**Chromatin signatures: DNaseI hypersensitivity and histone modification**. Average chromatin accessibility profiles and various histone modifications surrounding the TSS for coding genes, transcribed pseudogenes, and non-transcribed pseudogenes. The coding gene histone modification profiles around the TSS follow known patterns - for example, enrichment of H3K4me1 around 1 kb upstream of the TSS and the H3K4me3 peaks close to the TSS [63]. Transcribed pseudogenes also show stronger H3K4 signals than non-transcribed pseudogenes. H3K27me3, a marker commonly associated with gene repression [64], showed depletion around the TSS for the coding gene and a distinctive peak in the same region for the pseudogenes. H3K36me3 also shows a similar pattern as H3K27me3 at TSSs, which may relate to nucleosome depletion.

We compared the chromatin accessibility around the transcription start site (TSS) for active coding genes, transcribed and non-transcribed pseudogenes. DNaseI hypersensitivity signals along 8 kb regions surrounding the TSSs were averaged across all the genomic sequences in each of the three different groups. Transcribed pseudogenes show enhanced DNaseI hypersensitivity compared to non-transcribed pseudogenes on average, although, as expected, both signal profiles were lower than that for the coding genes (Figure 8).

A series of histone marks was also analyzed in the same manner as for the chromatin accessibility (Figure 8). In general, we found that the transcribed pseudogenes show more enhanced signals for active histone marks such as H3K4me1 and H3K4me3 than the non-transcribed pseudogenes, while they show little difference between the signals for repressive histone marks, such as H3K27me3. Our results show that, on average, the transcribed pseudogenes possess more transcriptional potential than non-transcribed ones, and their regulation mechanism may be similar to that of protein-coding genes.

#### Chromatin state segmentation

**Figure 9:**
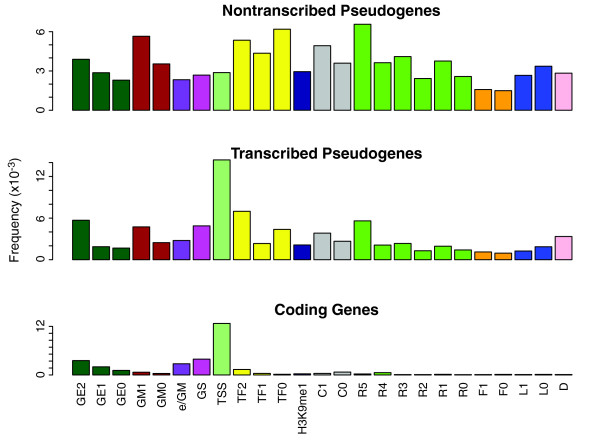
**Segmentation: comparison of chromatin segmentations associated with pseudogenes and parent genes**. The transcribed pseudogenes were selected based on the following criteria: there is transcription evidence from GENCODE, BodyMap or mass spectrometry studies; there is no known overlap with annotated coding genes; and there are no neighboring protein-coding gene TSSs 4 kb upstream or downstream of the pseudogene start.

There is a large variety of chromatin marks available. Therefore, we decided to use the chromatin states as a higher level feature in order to summarize all these descriptors. The chromatin states were assessed using the Segway segmentation pattern as defined by [48]. Segway annotates the genome using 25 different labels (Table S1 in Additional file 1) representing active and repressive marks. The genome-wide distribution of the segments shows a higher density of repressive markers compared to those indicating transcriptional activity. We analyzed the frequency of Segway markers for transcribed and non-transcribed pseudogenes, and their respective parent genes (Figure 9). We note that the non-transcribed pseudogenes show a depletion of TSS marks compared to transcribed pseudogenes, but enrichment in repressive marks. These results are in accordance with the trends noted earlier for histone modifications and chromatin accessibility.

**Figure 10:**
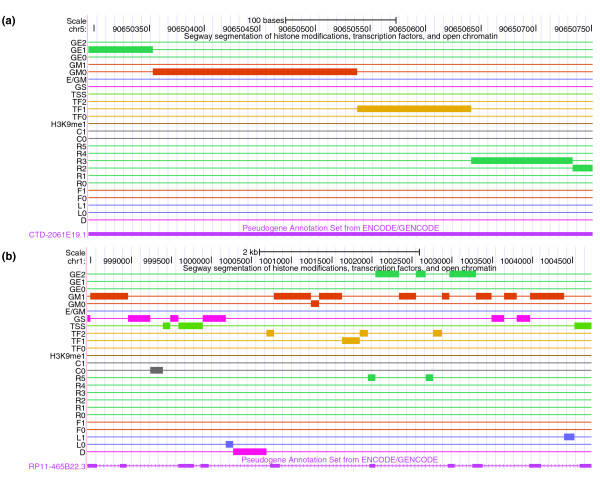
**Examples of pseudogenes with active chromatin states**. **(a) **Processed pseudogenes (Ensembl gene ID: ENST00000495909; genomic location chr5: 90650295-90650751). This pseudogene shows marks of activity based on segmentation-activity selection criterion 2. **(b) **Transcribed duplicated pseudogene (Ensembl gene ID: ENST00000412397.1; genomic location chr1: 998456-1004735). This pseudogene shows marks of activity based on segmentation-activity selection criterion 1.

The pattern of a high frequency of TSSs and gene body marks exhibited by the parent gene was considered a hallmark of active chromatin. Based on this observation, we developed a model using two selection criteria to pinpoint pseudogenes with active chromatin states: (1) the frequency of the TSS is three times higher than the frequency of any repressive markers; (2) the gene body start (GS), gene body middle (GM) and gene body end (GE) frequencies are two times larger than the frequency of the repressive markers. The selection criteria were chosen to match the segmentation behavior of the active genes. We identified 915 pseudogenes with active chromatin (92 using the first selection criterion and 823 using the second criterion) in the K562 cell line. Examples of pseudogenes with active chromatin states are shown in Figure 10. The pseudogenes selected using the above criteria are indicated in the psiDR for each cell line analyzed.

### Upstream regulatory elements

Given the importance of transcription in understanding pseudogene function and biological behavior, we focused our next analysis on the regulatory elements present in the upstream sequences of pseudogenes. More specifically, we investigated TFBSs, active RNA polymerase II (Pol2) binding sites and the active promoters of pseudogenes. All the information regarding the upstream regulatory elements of each pseudogene is recorded in psiDR.

#### Identification of transcription factor binding sites

We examined the TFBSs located in the upstream regions of all pseudogenes. A large fraction of pseudogenes contain no TFBSs in their upstream sequences (that is, 91.0%, 86.7%, 92.0%, 92.7% and 86.7% in Gm12878, K562, Helas3, H1-hesc and Hepg2 cell lines, respectively). This is consistent with the previous results showing most pseudogenes are not transcribed and have unfavorable chromatin structures.

**Figure 11:**
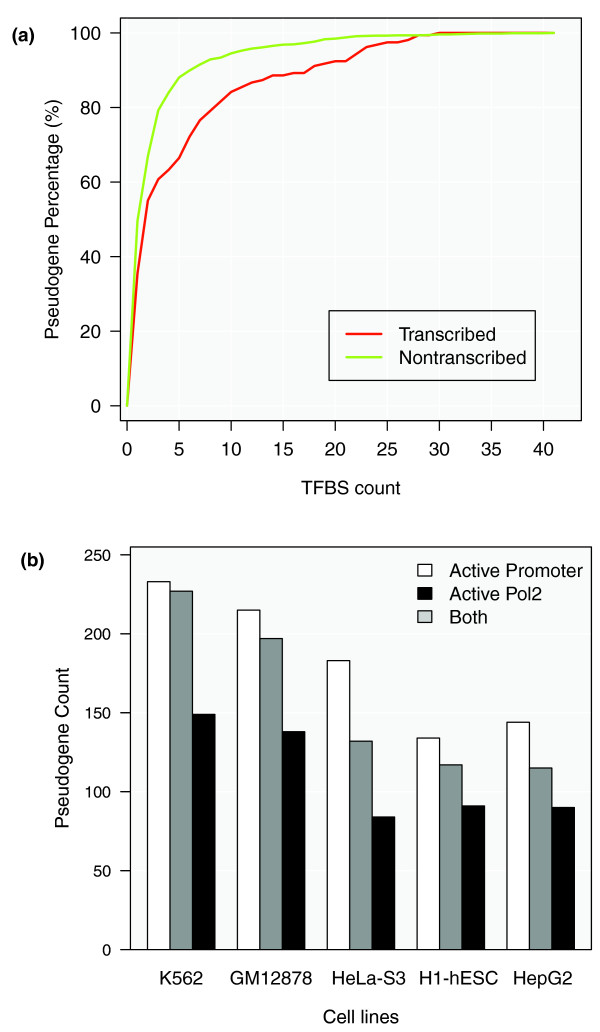
**Transcription factor binding sites upstream of pseudogenes**. **(a) **Distribution of pseudogenes with different numbers of TFBSs in their upstream sequences. Profiles from transcribed pseudogenes and non-transcribed pseudogenes are compared. Data are from the K562 cell line. **(b) **Number of pseudogenes with active promoters, active Pol2 binding sites or both in different cell lines.

Transcription factors that bind to the upstream regions of transcribed and non-transcribed pseudogenes were examined. Compared to the non-transcribed pseudogenes, the transcribed pseudogenes tend to have more TFBSs in the K562 cell line, although in both groups, the majority of pseudogenes contain no or very few (one or two) binding sites in their upstream regions (Figure 11). The difference between the number of TFBSs in the transcribed and non-transcribed pseudogenes is small but statistically significant (Wilcoxon rank-sum test, *P*-value = 3.8 × 10^-3 ^in K562). Similar results can be seen in the other four cell lines (Figure S7 in Additional file 1).

#### Pol2 binding sites

Pseudogenes were also examined in each cell line for potential Pol2 binding sites in their upstream sequences. To alleviate the potential mapping artifacts from the ChIP-Seq analysis, we applied a filter on Pol2 binding peaks to retain only the strong signals (see Materials and methods). Three selection criteria were used to identify pseudogenes with active Pol2 signals: (1) the width of a Pol2 binding peak is larger than the top 5% of all Pol2 peak widths across the ENCODE cell lines - the threshold based on ENCODE 2011 January freeze data is 519 bp; (2) the signal value of a Pol2 binding peak is larger than the top 5% of all Pol2 signal values across all the studied ENCODE cell lines - the threshold based on ENCODE 2011 January freeze data is 2.38; (3) at least one of the Pol2 cofactors included in the ENCODE project (Taf1, Taf7, Tbp, Nelfe, Gtf2f1, Gtf2b and Ccnt2) also binds to the upstream sequence of the pseudogene being studied.

A pseudogene that satisfied criteria 1 and 2 or satisfied criterion 3 was considered to have active Pol2 binding sites. In the K562, Gm12878, Helas3, H1hesc and Hepg2 cell lines, 227, 197, 132, 117 and 115 pseudogenes, respectively, have been shown to have active Pol2 binding sites. Active Pol2 binding sites were significantly enriched in the transcribed pseudogenes, where the *P*-values were 1.95 × 10^-9 ^(K562), 3.57 × 10^-13 ^(Gm12878), 7.38 × 10^-12 ^(Helas3), 3.24 × 10^-10 ^(H1hesc) and 1.96 × 10^-10 ^(Hepg2).

#### Active promoters for pseudogenes

We used the random forest model developed by Yip *et al*. [49] to predict active promoter regions for all the pseudogenes in each cell line. The objective of this model is to capture general properties of genomic regions, such as regulatory modules, by integrating approximately 500 ChIP-Seq experiments for more than 100 transcription and related factors. It calculates the likelihood of a region being an active promoter based on the chromatin accessibility data (from both DNase I hypersensitivity and FAIRE (formaldehyde-assisted isolation of regulatory elements) experiments), histone modifications, transcription factor binding, and conservation [49]. By intersecting the resultant set of active promoters from the model with pseudogene upstream sequences, we found that 233, 215, 183, 134, and 144 pseudogenes from K562, Gm12878, Helas3, H1hesc, and Hegp2 cell lines, respectively, possess active promoters. In all the cell lines, active promoters were significantly enriched in the transcribed pseudogenes, where the *P*-values were 1.19 × 10^-5 ^(K562), 1.95 × 10^-12 ^(Gm12878), 4.45 × 10^-10 ^(Helas3), 1.22 × 10^-11 ^(H1hesc) and 7.20 × 10^-12 ^(Hepg2).

### Data integration in psiDR

As shown in the previous sections, pseudogenes maintain diversified and complicated activity patterns, and the same pseudogene may exhibit different activities across different tissues. In this section, we will integrate the data in psiDR across a variety of partial activities.

#### Tissue specificity of pseudogene activities

First, we investigated the tissue specificity patterns observed for pseudogene transcription (Figure 5d). Among the 344 transcribed pseudogenes from the Illumina Human BodyMap data, 10 were transcribed in all the 16 tissues, while 190 were transcribed in one tissue only. Testis contained the largest number of transcribed pseudogenes (127 out of 344), and skeletal muscle contained the least (16 out of 344).

The pseudogenes with upstream regulatory regions - that is, active promoters and active Pol2 binding sites - also exhibit tissue specificity. We measured the similarity between any two active pseudogene sets from different cell lines with the Jaccard index, which is defined as the ratio of the size of the intersection divided by the size of the union of the two sets. The similarities of active pseudogenes between each pair of cell lines are summarized in Table 4. The values range from 0.22 to 0.39. The low similarity values between different cell lines indicate that these cells have distinct active pseudogenes.

**Table 4:** Similarity between pseudogenes with active promoters (upper right cells) and Pol2 binding sites (lower left cells)

Cell line	K562	Gm12878	Helas3	H1hesc	Hepg2
K562	-	0.30	0.29	0.22	0.27
Gm12878	0.33	-	0.33	0.27	0.32
Helas3	0.31	0.31	-	0.30	0.39
H1hesc	0.24	0.27	0.29	-	0.27
Hepg2	0.26	0.32	0.33	0.33	-

We also examined the transcription factors whose binding sites were enriched in the transcribed pseudogenes compared to the non-transcribed pseudogenes. Some general-purpose factors such as Pol2 were enriched in transcribed pseudogenes of all the cell lines, while each cell line also had some unique transcription factors (Table S2 in Additional file 1). In some cases, the transcription factors unique to a cell line were found to be associated with the biological roles of that cell. For example, Hnf4a, which is a nuclear transcription factor with a role in liver development, was only enriched in active pseudogenes in the liver cell line Hepg2, while Pou2f2, which activates immunoglobulin gene expression, was only enriched in active pseudogenes in the B-lymphocyte cell line Gm12878.

#### Overall degree of partial activity

**Figure 12:**
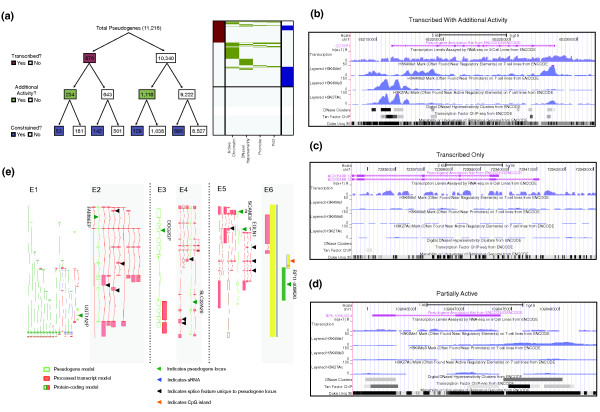
**Summary of pseudogene annotation and case studies**. **(a) **A heatmap showing the annotation for transcribed pseudogenes including active chromatin segmentation, DNaseI hypersensitivity, active promoter, active Pol2, and conserved sequences. Raw data were from the K562 cell line. **(b) **A transcribed duplicated pseudogene (Ensembl gene ID: ENST00000434500.1; genomic location, chr7: 65216129-65228323) showing consistent active chromatin accessibility, histone marks, and TFBSs in its upstream sequences. **(c) **A transcribed processed pseudogene (Ensembl gene ID: ENST00000355920.3; genomic location, chr7: 72333321-72339656) with no active chromatin features or conserved sequences. **(d) **A non-transcribed duplicated pseudogene showing partial activity patterns (Ensembl gene ID: ENST00000429752.2; genomic location, chr1: 109646053-109647388). **(e) **Examples of partially active pseudogenes. E1 and E2 are examples of duplicated pseudogenes. E1 shows *UGT1A2P *(Ensembl gene ID: ENST00000454886), indicated by the green arrowhead. *UTG1A2P *is a non-transcribed pseudogene with active chromatin and it is under negative selection. Coding exons of protein-coding paralogous loci are represented by dark green boxes and UTR exons by filled red boxes. E2 shows *FAM86EP *(Ensembl gene ID: ENST00000510506) as open green boxes, which is a transcribed pseudogene with active chromatin and upstream TFBSs and Pol2 binding sites. The transcript models associated with the locus are displayed as filled red boxes. Black arrowheads indicate features novel to the pseudogene locus. E3 and E4 show two unitary pseudogenes. E3 shows *DOC2GP *(Ensembl gene ID: ENST00000514950) as open green boxes, and transcript models associated with the locus are shown as filled red boxes. E4 shows *SLC22A20 *(Ensembl gene ID: ENST00000530038). Again, the pseudogene model is represented as open green boxes, transcript models associated with the locus as filled red boxes, and black arrowheads indicate features novel to the pseudogene locus. E5 and E6 show two processed pseudogenes. E5 shows pseudogene *EGLN1 *(Ensembl gene ID: ENST00000531623) inserted into duplicated pseudogene *SCAND2 *(Ensembl gene ID: ENST00000541103), which is a transcribed pseudogene showing active chromatin but no upstream regulatory regions as seen in the parent gene. The pseudogene models are represented as open green boxes, transcript models associated with the locus are displayed as filled red boxes, and black arrowheads indicate features novel to the pseudogene locus. E6 shows a processed pseudogene *RP11*-*409K20 *(Ensembl gene ID: ENST00000417984; filled green box), which has been inserted into a CpG island, indicated by an orange arrowhead. sRNA, small RNA.

A graphical overview of pseudogene activity data included in psiDR for cell line K562 is plotted in Figure 12a. Additional activity of pseudogenes (beyond transcription) was obtained from one or more of the statistical models for chromatin state, chromatin accessibility, Pol2 binding and upstream promoter regions, as discussed in the previous sections. It can be seen that pseudogenes form a diversified group, where there are very few pseudogenes showing consistently active signals across all the biological features and many showing little or no activity.

It is interesting to note that there are pseudogenes showing all kinds of partial activity (examples in Figure 12b-e). Comparing the pseudogene features indicative of genomic activity with their parent gene counterparts, we noticed a number of interesting cases.

There are 13 non-transcribed pseudogenes in K562 cell with active chromatin that have retained the upstream regulatory regions of the parent gene and are under strong negative selection. Collectively, these features suggest that these pseudogenes are representative of 'dying' genes, which may have recently lost their transcription activity and are in the process of losing functionality. The *UGT1A2P *duplicated pseudogene is representative of this class (E1 in Figure 12e). It is still under selective constraint and appears to be well positioned for transcription and the production of a full-length transcript, lying proximal to active paralogs; however, it does not exhibit any transcriptional evidence. This apparent loss of features (transcription, splice donor) appears to support the hypothesis that this duplicated pseudogene is losing its function.

Conversely, there are examples of transcribed pseudogenes showing signals of active chromatin, DNaseI hypersensitivity, active promoter, and Pol2 binding sites, which appear to be gaining new functionality. A good example is *FAM86EP *(E2 in Figure 12e). The locus has gained five splice junctions (one acceptor and four donors), which suggest the possibility of new functionality being explored. There are other examples of transcribed pseudogenes with active chromatin but without retention of any of the parent gene's upstream elements. Changes in the sequences and the upstream regulatory elements can give rise to new transcript structures, resulting in a locus now encoding a ncRNA rather than a translated protein product. We hypothesize that these may be dead protein genes being 'resurrected' as ncRNAs. Two genes supporting this hypothesis are shown in Figure 12e (E5 and E6). E5 in Figure 12e shows pseudogene *EGLN1*, which has gained chromatin activity and active promoter signals via its insertion into a transcribed duplicated pseudogene locus (*SCAND2*). The combined locus is transcribed and its transcripts are subject to alternative splicing, with some transcripts incorporating sequence from both pseudogenes and having seven novel splice features (four acceptors and three donors). The novel pseudogene shown in E6 in Figure 12e appears to have gained transcriptional signals via its insertion proximal to a CpG island, which also supports the transcription of a lncRNA on the opposite strand.

In light of these examples, we believe that the partial activity patterns are reflective of the pseudogene evolutionary process, where a pseudogene may be in the process of either resurrection as a ncRNA or gradually losing its functionality. Understanding why pseudogenes show partial activity may shed light on pseudogene evolution and function.

## Discussion

### Pseudogene annotation

In this study, we describe a set of human pseudogenes at the genome-wide scale. The pseudogene dataset is created by manual annotation with the assistance of computational pipelines. The surveyed set of 11,216 consensus pseudogenes is the first comprehensive effort of manual annotation of human pseudogenes at the whole genome level.

### Pseudogenes and their parents

We combined manual annotation and sequence identity data to identify parent genes for approximately 86% of pseudogenes (9,636 out of 11,216). The numbers of protein-coding genes associated with pseudogenes is not evenly distributed: some housekeeping genes, such as those encoding ribosomal proteins and GAPDH, are among the parents having the most pseudogenes.

The sequence identity between pseudogenes and their parents is of interest for studies of pseudogene evolution and regulatory function. We found a unimodal distribution of sequence similarity between processed pseudogenes and parents, which reflects a recent burst of processed pseudogenes in human evolutionary history (Figure 4). In contrast, the uniform distribution of sequence similarity between duplicated pseudogenes and parents indicates that the duplication process is random and happens at a stable rate during genome evolution.

### Pseudogene transcription and tissue specificity

Several recent studies have highlighted the fact that pseudogenes can play active roles through their RNA products [50]. Using a large variety of biological data and statistical models, we predict that at least 9% of the pseudogenes present in the human genome are actively transcribed. We observed that although there are more processed pseudogenes than duplicated pseudogenes (8248 versus 2,127) in the human genome, the ratio between them is not maintained in the transcribed ones (520 versus 343). The duplicated pseudogenes are significantly enriched in the transcribed list (*P*-value close to 0). This is expected since the duplicated pseudogenes may retain the promoter regions of their parents when duplicated, unlike the processed pseudogenes that insert randomly into the genome and therefore require the presence of potential regulatory sequences in the neighboring genomic locations.

#### Pseudogene conservation

High sequence identity between pseudogenes and their parents does not necessarily imply selection pressure on the former since it can be due to recent pseudogenization events where a pseudogene has yet to accumulate mutations from neutral drift. Therefore, to better understand selection pressure on pseudogenes, we compared the pseudogene CDS and 3' UTR sequence identity to their corresponding parent regions. Sequence analysis highlights a group of pseudogenes showing differential evolutionary pressure on the two regions. Furthermore, analysis of human polymorphism data and pseudogene conservation shows a potential weak signal for selection on transcribed pseudogenes. Overall, we identify a number of pseudogenes under evolutionary constraint. Combined with transcription data, this list contains pseudogenes with potential biological function and may act as a good reference for additional experimental analysis.

### Partial activity of pseudogenes

We have integrated a large amount of genome-wide functional genomics data, together with expression and conservation data, to create a pseudogene annotation resource, psiDR. This allows us to comprehensively examine pseudogene activity from different perspectives, such as transcription, regulation and evolution. We found a number of pseudogenes showing activity and, more interestingly, a group of pseudogenes exhibiting various ranges of partial activity. Partially active pseudogenes were defined by a series of simple models based on transcription evidence, chromatin state, DNaseI hypersensitivity, upstream regulatory elements, and selection pressure. Different combinations of those features led to the characterization of pseudogenes as being partially active. One can speculate that partial activity may correspond to the process of resurrection of a pseudogene as a ncRNA or that it is in the process of dying and losing function. We believe that the various partially active pseudogenes provide a rich informative resource to aid understanding of pseudogene function and evolution.

One of the key aspects in defining the partially active pseudogenes is their upstream regulatory region. The presence or absence of regulatory elements is essential to understanding the evolutionary stage of the partially active pseudogenes. For example, a pseudogene showing active promoters and TFBSs but lacking transcription evidence is believed to be a 'dying' gene, while a pseudogene with markedly different upstream elements compared to its parent gene but showing evidence of transcription is regarded as being potentially 'resurrected'. In the present paper we define the partially active pseudogenes based on several genomic features: TFBSs, histone marks, DNA accessibility, and so on. However, we expect that future functional genomics datasets will complete the activity profiles of pseudogenes. In particular, integration of DNA methylation, nucleosome positioning, chromatin interaction analysis by paired-end tag sequencing (ChIA-PET), and high-throughput sequencing of RNA isolated by crosslinking immunoprecipitation (HITS-CLIP) datasets will provide a useful addition to the ENCODE pseudogene resource.

In conclusion, by integrating GENCODE pseudogene annotation, extensive functional genomics data from ENCODE and the variation data from the 1000 Genome project, we provide a comprehensive resource for pseudogene annotation and activity in the human genome. This resource has allowed us to classify pseudogenes with various attributes, which will enable interested researchers to identify expressed pseudogenes with potential function. Recent studies have shown the various ways by which pseudogenes regulate the expression of protein-coding genes and underscored the importance of identifying functional pseudogenes. We believe this resource provides data that can be used to further research in this direction. In particular, it is useful for understanding the regulatory role of pseudogenes, especially in cancer and other developmental processes. The comprehensive annotation of human pseudogenes also allows their comparison with pseudogenes from other model organisms, such as mouse, worm, fly, and cress, which can provide valuable information on genome evolution.

## Materials and methods

### Manual annotation

The manual annotation is based on protein data from the UniProt database, which is aligned to the individual bacterial artificial chromosome (BAC) clones that make up the reference genome sequence using BLAST [51]. Gene models are manually extrapolated from the alignments by annotators using the ZMAP annotation interface and the otterlace annotation system [52]. Alignments were navigated using the Blixem alignment viewer [53]. Visual inspection of the dot-plot output from the Dotter tool [53] is used to resolve any alignment with the genomic sequence that is unclear in, or absent from, Blixem. A model is defined as a pseudogene if it possesses one or more of the following characteristics unless there is evidence (transcriptional, functional, publication) showing that the locus represents a protein-coding gene with structural/functional divergence from its parent (paralog): (1) a premature stop codon relative to parent CDS - can be introduced by nonsense or frame-shift mutation; (2) a frame-shift in a functional domain - even where the length of the resulting CDS is similar to that of the parent CDS; (3) a truncation of the 5' or 3' end of the CDS relative to the parent CDS; (4) a deletion of an internal portion of the CDS relative to the parent CDS. Processed pseudogene loci lacking disabling mutations are annotated as 'pseudogene' when they lack locus-specific transcriptional evidence

### PseudoPipe

PseudoPipe identifies pseudogenes by searching for homology to all known protein sequences in the genome (defined in Ensembl) using a six-frame translational BLAST, followed by removal of redundancies and merging of the overlapping and continuous BLAST hits. Functional paralogs (parents) of the resulting pseudogenes are determined by sequence similarity, and the disablements in pseudogenes are identified through alignment to the parent genes. A non-redundant set of 18,046 pseudogenes was obtained using the human reference genome (GRch37, ENSEMBL gene release 60). Pseudogenes are categorized into different classes as processed, duplicated or ambiguous based on their genomic structures. While duplicated pseudogenes have intron-exon like structures, processed pseudogenes contain only continuous exon sequences with no introns and have traces of polyadenine tails at the 3' end. Ambiguous pseudogenes indicate processed pseudogenes with decayed sequences.

### RetroFinder

RetroFinder is unique among pseudogene prediction methods for using mRNA alignments to identify retrogenes, including processed pseudogenes [37]. Human mRNA and RefSeq sequences are aligned using the Lastz [54] alignment program (based on Blastz [55]), which is very sensitive, allowing alignment down to the level of 65% identity, whereas BLAT [56] works better for sequences where identity is greater than 95%. If one of these transcripts aligns more than once, and one of the alignments is to a known gene locus, then the additional alignments are scored on a number of features indicative of retrotransposition: multiple contiguous exons with the parent gene introns removed; negatively scored introns that are distinguished from repeat insertions (SVA elements, long interspersed nucleotide elements (LINEs), short interspersed nucleotide elements (SINEs), Alu elements); lack of conserved splice sites; break in synteny with mouse and dog genomes using the syntenic net alignments [57] from the UCSC Genome Browser [58]; polyadenine tail insertion.

Parents based on immunoglobulin and zinc finger genes are filtered out since these large gene families cause false positives. The score threshold is set at 550 based on training with VEGA [59] processed pseudogenes. Note that for human, VEGA genes are included in the manually annotated genes of GENCODE. Further details of the method can be found in [37].

### Consensus of manual and automated annotation

To obtain a consensus set of pseudogenes, we verified each pseudogene locus from manual annotation against those predicted by either of the two automated pipelines (PseudoPipe and RetroFinder), using a 50 bp overlap criterion. A pseudogene passing these overlapping tests is classified as: a 'level 1' pseudogene if it passes tests of manual annotation against both automated pipelines; or a '2-way consensus' pseudogene if it only passes the test between the two automated pipelines.

As a quality control exercise to determine completeness of pseudogene annotation in chromosomes that have been manually annotated, 2-way consensus pseudogenes are re-checked to establish their validity and added to the manually annotated pseudogene set as appropriate.

### Pseudogene extrapolation

We estimated the total number of pseudogenes in the genome using the knowledge from PseudoPipe and manual annotation. Using manual annotation from the chromosomes that were completely annotated as a gold standard, we estimated the number of false positives and false negatives in PseudoPipe predictions. We used this information to extrapolate to the entire human genome to obtain an estimate of the number of pseudogenes in the reference genome.

Chromosomes 1 to 11, 20, 21, 22, X, Y and the p arm of 12 are fully annotated in GENCODE v7. On these chromosomes, there are 9,776 and 12,501 pseudogenes predicted by manual inspection and by PseudoPipe, respectively. PseudoPipe assigned 18,046 pseudogenes in the entire genome. Based on this, the number of manually identified pseudogenes in the genome will be (9,776 × 18,046)/12,501 ≈ 14,112.

Alternatively, we used a simple linear extrapolation to correlate the number of pseudogenes with the size of chromosomes on which the pseudogenes are annotated. With this method, the number of nucleotides from the fully annotated regions is 2,383,814,825, while the total number of nucleotides in the genome is 3,092,688,347. Therefore, the predicted number of pseudogenes for the entire human genome is (9,776 × 3,092,688,347)/2,383,814,825 ≈ 12,683.

### Identification of the parents of pseudogenes and sequence similarity to the parent

We derived parents of pseudogenes from the correspondence between pseudogenes and query sequences used by different pipelines (that is, UniProt proteins for manual annotation and Ensembl peptides for PseudoPipe), together with the sequence alignments of pseudogenes against the whole human genome. The procedure was carried out using the following steps: first, use correspondence between parents and pseudogenes derived by the manual annotation; second, one-to-one sequence alignment between pseudogenes and coding regions in the human genome by BLAT (sequence similarity > 90%); third, use parent gene information provided by PseudoPipe.

When the parent identity for a pseudogene is inconsistent across different data resources, we assign the parent based on the highest ranked data in the following order: manual annotation, BLAT alignment, and automated curation.

Parents of 9,368 pseudogenes were unambiguously identified, while it is difficult to uniquely identify the parent genes for 1,848 pseudogenes. The two most significant factors that confound our ability to confidently identify a pseudogene parent are the degree of degradation of the pseudogene and the number of closely related paralogs to the true parent gene. Therefore, for gene families with many closely related members, even a relatively small number of mutations can render accurate identification of the true parent difficult; while for more degraded pseudogenes from large families with common functional domains (for example, zinc fingers), the number and similarity of the potential parents make prediction impossible.

To calculate the sequence identity between pseudogenes and their parents, each pseudogene sequence was extended by 2 kb at its 3' end for a higher coverage of 3' UTR of its parent and then aligned to its parent sequence. Only exons of parent and pseudogene sequences were used. The alignment was carried out using ClustalW2, with default parameters. To adapt to the large size of 3' UTR and much smaller size of small RNA targets in that region, a sliding window of 100 bp was used for sequence identity for a more accurate local identity. The window with the highest sequence identity was taken as representative of the 3' UTR and used in the following tests.

### Pseudogene transcription evidence from RNA-Seq data

The pseudogenes in GENCODE v7 were tested for transcription evidence using the following workflow. First, we extracted the genomic coordinates of the processed and duplicated pseudogenes from GENCODE v7 (gene_type = 'pseudogene' AND transcript_type = 'processed_pseudogene' OR transcript_type = 'unprocessed_pseudogene'). From this step we obtained 8,107 processed and 1,860 duplicated pseudogenes. Second, we obtained the underlying genomic sequence for each pseudogene by concatenating the sequences of their pseudoexons. Third, we aligned each pseudogene sequence to the human reference genome using BLAT [56] (with default parameters) to find all similar regions in the genome. Fourth, we assigned each pseudogene alignment to one of four categories: pseudogenes with no similar regions in the genome (presumably these pseudogenes are more ancient and have accumulated many mutations, and therefore they have a low sequence similarity compared to the parent gene); pseudogenes giving rise to one alignment pair (most likely the parent gene); pseudogenes with two to five alignments; pseudogenes giving rise to more than five sequence alignments.

For the 9,967 pseudogenes analyzed, we obtained the following counts: 3,198 pseudogenes with zero alignments, 1,907 pseudogenes with one alignment, 2,150 pseudogenes with two to five alignments and 2,712 pseudogenes with more than five alignments.

In order to check for evidence of pseudogene transcription, we examined the expression pattern of each pseudogene and its similar regions using the Illumina Human BodyMap RNA-Seq data set consisting of 16 tissues. First, we aligned the reads for each tissue to the human genome reference sequence in conjunction with a splice junction library using Bowtie [60] and RSEQtools [61]. There was no preference given for a genome match over other matches. Second, we generated a signal track of the mapped reads for each tissue. Third, for a given pseudogene and its similar regions in the human genome, we extracted the signal track of mapped reads from all 16 tissues as shown in Figure 5c.

After a number of filtering steps we obtained a list of potentially transcribed pseudogenes. For example, the set of 3,198 pseudogenes with no similar regions in the genome was reduced to 344 pseudogenes by requiring that each pseudogene is covered by at least two reads across half of its length in at least one tissue.

### Transcribed pseudogenes subject to experimental validation

Out of the 469 pseudogenes subjected to experimental validation, 94 pseudogenes were randomly selected from the manual pipeline output (pipeline 1 in section '*Pseudogene Transcription Identified by Sequence of Computational Pipelines'*); 271 pseudogenes were selected at random from the PseudoSeq pipeline output (pipeline 3 in the same section as above), and 97 pseudogenes were selected at random from the TotalRNA pipeline output (pipeline 2 in the same section as above). The remaining seven pseudogenes (containing seven loci to be validated), were manually chosen by examining the expression patterns of pseudogenes and their parents using BodyMap data and PseudoSeq (Figure 5c). At the time of writing, the remainder of transcribed pseudogenes are undergoing experimental validation and the results will be constantly updated in the psiDR.

### Multiple sequence alignment, pseudogene preservation and polymorphisms in the human population

#### Sequence alignment

Genomic sequence alignments of 16 species, including primates, mammals, and vertebrates, were extracted from the original 46-way vertebrate sequence alignments obtained from the UCSC genome browser. Genomes from all the species were aligned using BlastZ with a synteny filter followed by the MultiZ method. Assembled sequences for the 2X mammal data are excluded from the current study due to their low quality and possible false positive alignment to pseudogenes from the high-quality assemblies.

#### Selection pressure

Genomic variation data consisting of SNPs, indels, and structural variations were from 60 individuals in the CEU population (Utah residents with ancestry from northern and western Europe) from the 1000 Genomes project pilot data release [47].

#### Pseudogene conservation

Chimp orthologs to human pseudogenes were derived from whole genome sequence alignments. Only pseudoexons were used in the ortholog identification and the following analyses. The divergence is calculated as the ratio of mutated nucleotides in the chimp genome to the length of human pseudogenes. We assume the occurrence of substitution follows a Poisson distribution and the background substitution rate (null hypothesis mean) was set at 1.5%. The *P*-value for pseudogene conservation was derived as the probability of that pseudogene having equal or fewer nucleotide mutations than it really has under the null hypothesis. We adjusted *P*-values for multiple hypotheses testing using the Benjamini and Hochberg approach [62]. All the pseudogenes were ranked by their *P*-values from the most significant to the least significant. Pseudogenes with *P*-values less than (False discovery rate × Rank/COUNT) were taken as significant, where false discovery rate is set to 0.05 and COUNT is the total number of pseudogenes tested. Conserved pseudogenes from mouse orthologs were calculated in the same manner, except the background substitution rate was set to 5%.

### Chromatin segmentation using segway

Segway segmentation labels the genome using 25 different markers. Half of them are indicative of genomic activity (for example, transcription factor activity, gene body, enhancers), while the other half are repressive (for example, CTCF). We calculated the frequency of each marker in the pseudogenes and parent genes in a genome-wide fashion. All the frequencies were normalized with respect to the total segment distribution across the entire genome. Two different trends were observed globally for the parent genes: (a) TSS mark frequency is at least one order of magnitude larger than the frequency of the repressive marks; and (b) the frequency of the GE, GM and GS marks is, on average, five times larger than the frequency of the repressive marks. The segment distribution of the parent genes indicated enrichment in TSS, GS, e/GM (enhancer/gene body middle) and GE marks and was considered as a standard indicator for active chromatin.

### Transcription factor binding sites in the upstream regions

TFBSs were studied using data from ENCODE ChIP-Seq experiments. In this study, we used the transcription factor occupancy data from the ENCODE 2011 January data freeze. The binding peaks of all the transcription factors were called by PeakSeq, with optimal settings to reduce the false negative results due to weak/poor biological replicates. A pseudogene was considered to have a TFBS if the majority of a peak for that transcription factor is located within the genomic region 2 kb upstream of the pseudogene.

ENCODE tier 1 and tier 2 cell lines (Gm12878, K562, Helas3, H1-hesc and Hepg2) with ChIP-Seq data for at least 40 transcription factors were included in this analysis. To avoid confusion with the transcription factor binding signals from neighboring genomic loci, 693 pseudogenes whose 5' ends are less than 4 kb away from the TSS of protein-coding genes were excluded. In the end, this study focused on 10,523 pseudogenes, where 876 are transcribed pseudogenes.

One confounding factor in the analysis is the different number of transcription factors studied in each cell line. However, we argue that the numbers here reflect the true tendency of TFBSs for pseudogenes since fairly comprehensive lists of transcription factors have been studied (74, 114, 53, 40 and 61 transcription factors in Gm12878, K562, Helas3, H1-hesc and Hepg2, respectively) and the results are consistent across all the different cell lines.

## Abbreviations

bp: base pair; CDS: coding sequence; ChIP: chromatin immunoprecipitation; EST: expressed sequence tag; GE: gene body end; GM: gene body middle; e/GM: enhancer/gene body middle; GS: gene body start; HAVANA: Human and Vertebrate Analysis and Annotation; lncRNA: long non-coding RNA; miRNA: microRNA; ncRNA: non-coding RNA; Pol2: RNA polymerase II; psiDR: Pseudogene Decoration Resource; RT-PCR: reverse transcription polymerase chain reaction; siRNA: small interfering RNA; SNP: single nucleotide polymorphism; SV: structural variants; TFBS: transcription factor binding site; TSS: transcription start site; UCSC: University of California at Santa Cruz;UTR: untranslated region.

## Competing interests

The authors declare that they have no competing interests.

## Authors' contributions

BP, CS and MG conceived of the study. BP carried out pseudogene parent analysis and upstream analysis, participated in chromatin signature and pseudogene conservation studies and drafted the manuscript. CS carried out chromatin segmentation studies and participated in pseudogene parent analysis and revision of the manuscript. AF, TH and JH carried out the manual annotation of pseudogenes. CH, AT and AR performed the experimental validation of transcribed pseudogenes. LH carried out the automated identification of transcribed pseudogenes. XJM carried out the evolutionary selection studies. RH and MD participated in the pseudogene annotation. SB participated in pseudogene annotation and revision of the manuscript. MG participated in revision of the manuscript and coordinated the whole study. All the authors read and approved the final manuscript.

## Supplementary Material

Additional file 1 **Supplementary tables and figures**. Click here for file
