# Supplementary material for: The GENCODE pseudogene resource
Source: Genome Biol. 2012 Sep 5;13(9):R51. doi: 10.1186/gb-2012-13-9-r51 (PMC3491395; doi:10.1186/gb-2012-13-9-r51)
Supplement: Additional file 1 — Supplementary tables and figures. [file gb-2012-13-9-r51-S1.pdf]

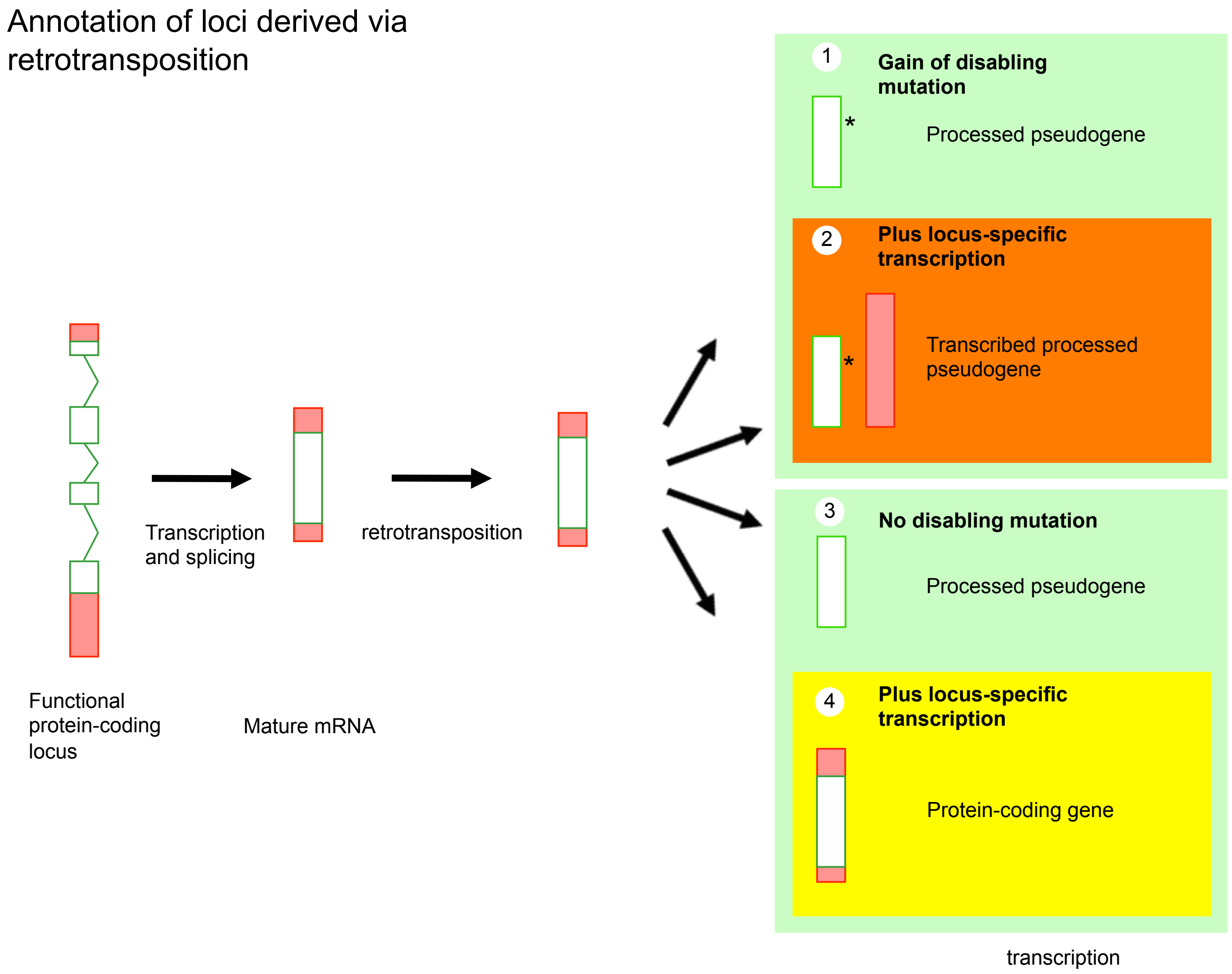

Figure S0: Schematic illustration of the annotation of loci derived via retrotransposition. Loci are identified as having arisen via retrotransposition primarily by identifying changes to their gene structure relative to closely related paralogous loci, specifically, loss of exon-intron structure as a result of the removal of introns in the 'parental' transcript via splicing. Following its creation by insertion of a spliced transcript into the genome, the locus is subject to one of four possible broad fates which is reflected in the way it is represented in the Gencode geneset. Where a locus gains a mutation likely to be disabling it is annotated as either a processed_pseudogene (1) or, where locus specific transcriptional evidence can be identified, as a transcribed_processed_pseudogene (2). Where a locus does not gain a disabling mutation and there is no evidence of locus specific transcriptional evidence it is annotated as a processed_pseudogene (3); where locus-specific transcriptional evidence is present and the CDS does not contain a disabling mutation, the locus is annotated as protein_coding (4).

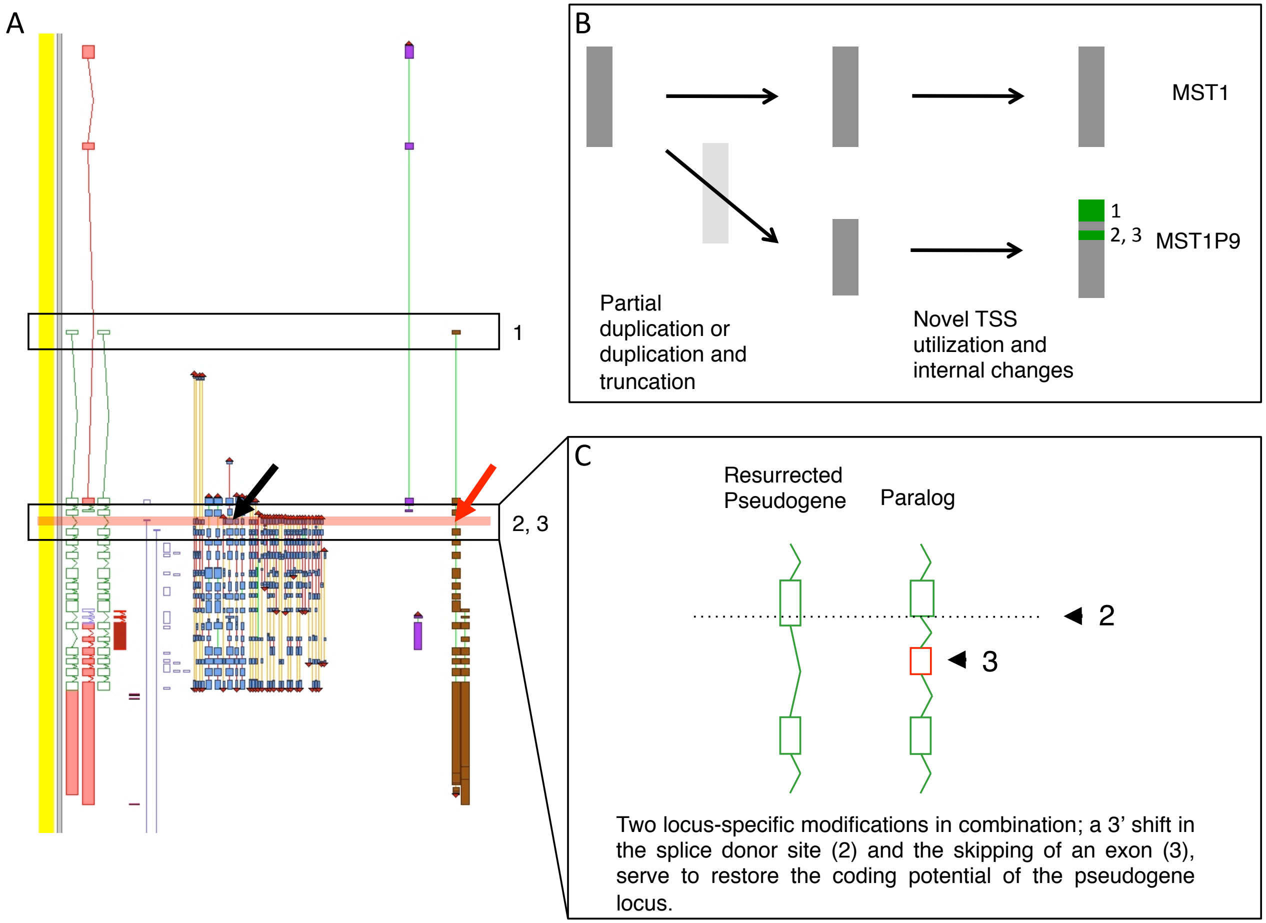

Figure S1: Difficulties in pseudogene annotation. Case study - pseudogene resurrection. (A) MST1P9 locus as viewed in the Zmap manual annotation interface. UTR exons and splice variants with no annotated CDS are shown in red, coding exons are shown in green and the CDS portion of models annotated as NMD are shown in purple. The upper boxed section shows the novel 5' end and the lower boxed section highlights the part of the model critical to the coding potential of the locus while the red bar highlights the position of the exon whose incorporation introduces the disablement that pseudogenises the locus. The alignment of protein sequences (pale blue boxes) from orthologous and paralogous loci can be clearly seen to incorporate this exon (indicated by black arrow). Locus-specific transcript evidence is shown to the right of the figure; EST evidence is represented as purple boxes and mRNA evidence in brown. The red arrow highlights the alternative structure of the full-length mRNA (AY192149), which supports the annotation of a coding gene model with a full-length CDS. (B) Schematic of the proposed origin of the MST1P9 locus. MST1P9 is derived from the MST1 almost certainly via the intermediate locus (MST1P2) but it is 5' truncated relative to both paralogous loci. MST1P9 has acquired a novel TSS and 5' exons leading to translation initiation at a different AUG (1) and two internal changes (2,3) required to allow the translation of a complete CDS. (C) Details of the internal changes. Transcripts from the MST1P9 locus utilize a downstream splice donor which is never used by transcripts from the MST1 locus, and this shifted splice junction, in combination with the skipping of the adjacent downstream exon is sufficient to restore a full-length CDS at the MST1P9 locus.

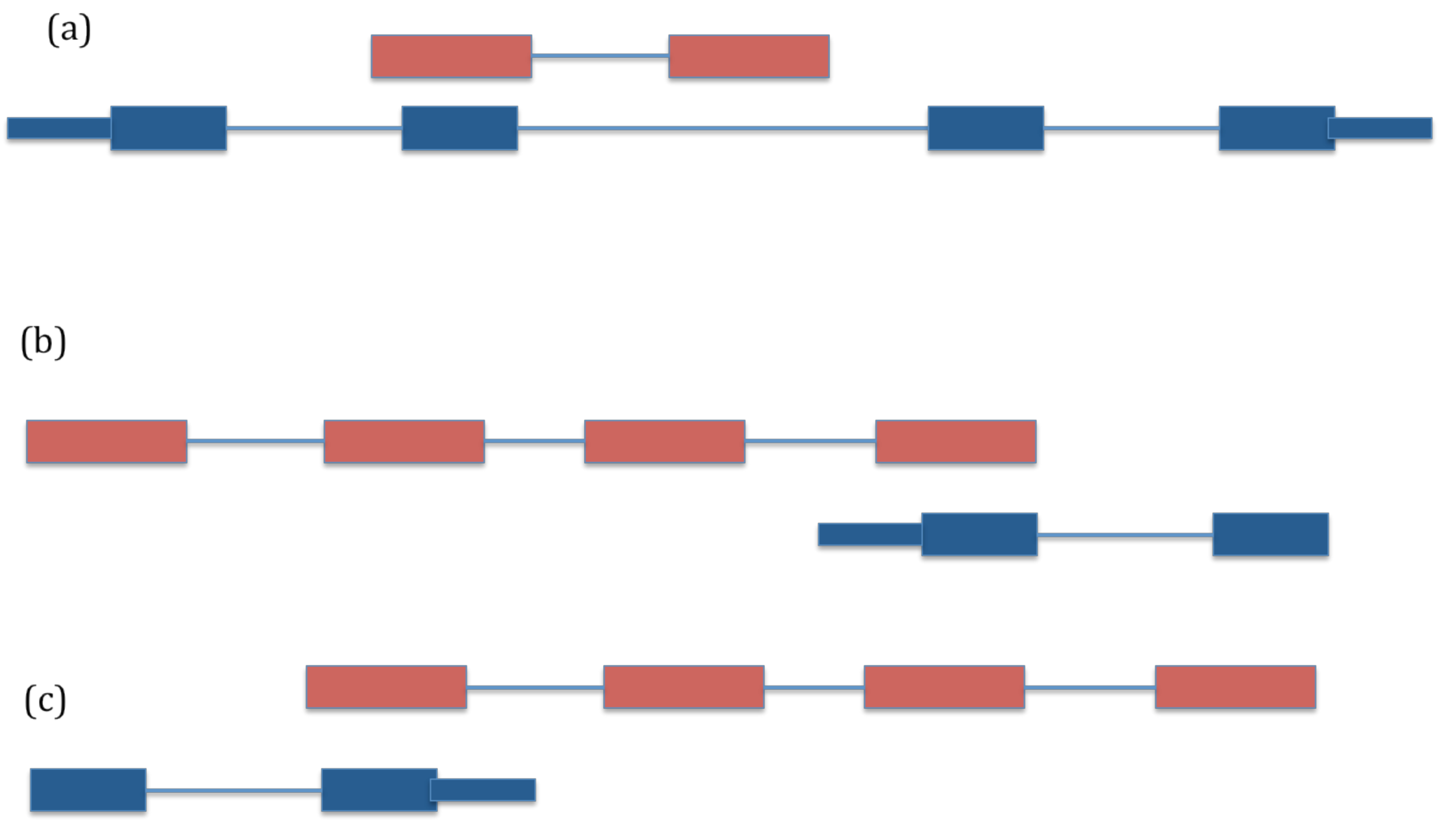

Figure S2: Pseudogenes overlapping with protein coding genes. (a) Part of the pseudogene sequence is used to create a new alternatively spliced internal exon in the protein-coding gene. (b) The pseudogene sequence contributes the 5’ terminal exon of the protein-coding gene. (c) The pseudogene sequence contributes the 3’ terminal exon of the protein-coding gene.

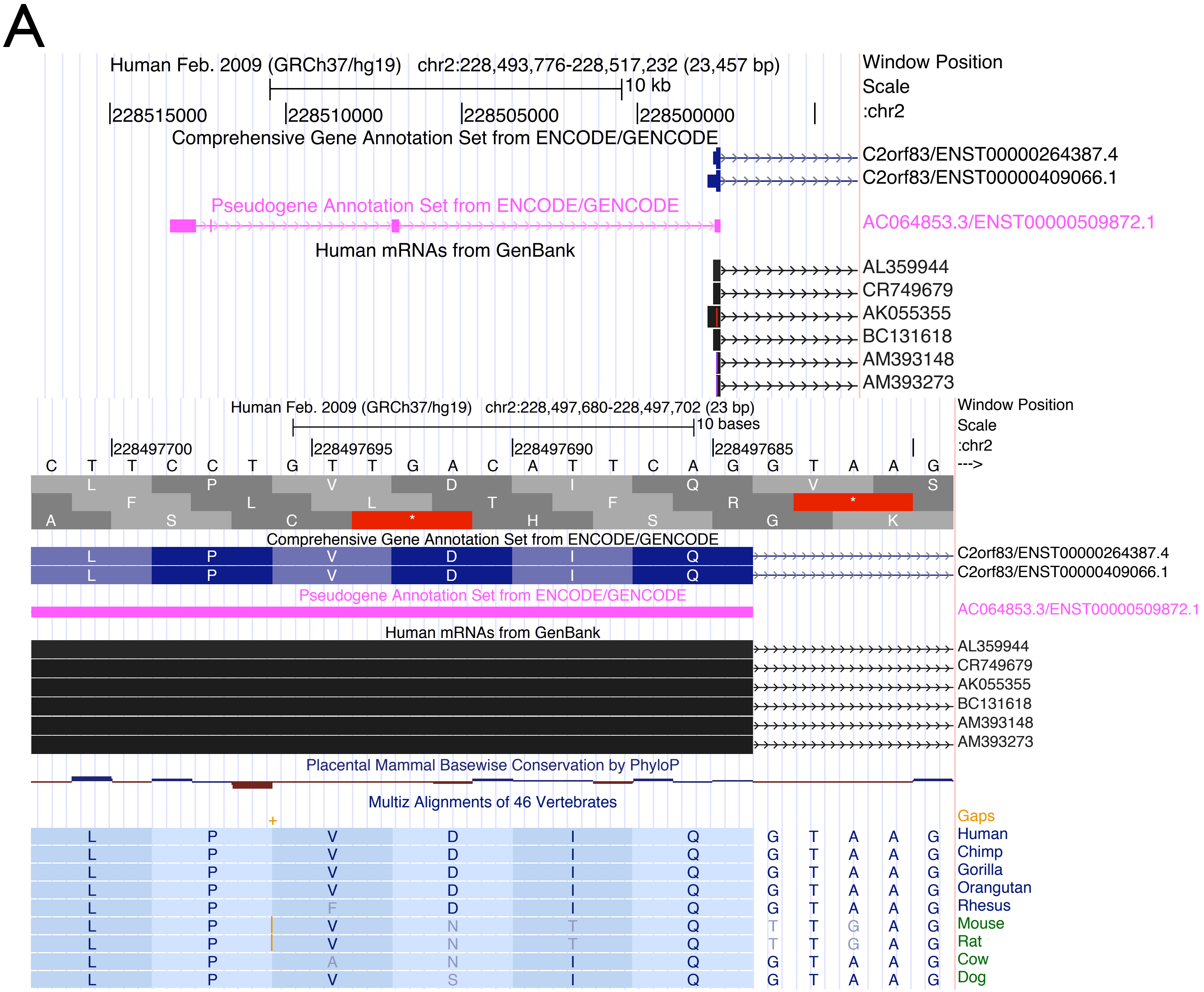

Figure S3(A): Examples of pseudogenes overlapping with protein-coding genes. Unprocessed pseudogene and protein-coding annotation overlap on same strand. Solute Carrier Family 19, member 3 (SLC19A3) pseudogene (AC064853.3) ends at end of first coding exon on coding transcript.

B

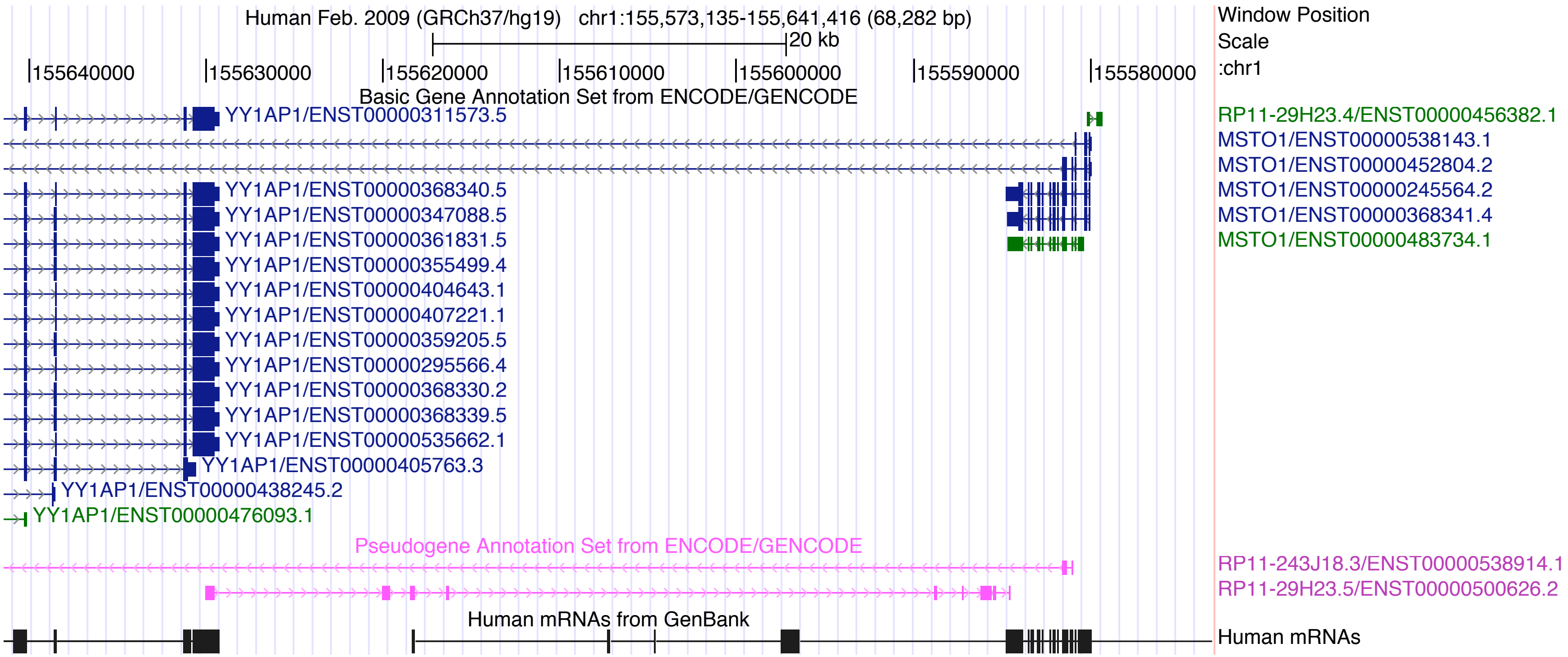

Figure S3(B): Examples of pseudogenes overlapping with protein coding genes. Pseudogene and protein coding annotation overlap on different strands. Novel Pseudogene - RP11-29H23.5-001 overlaps the coding part of the final exon of YY1AP on the same strand and the 3' UTR of MSTO1 on the opposite strand.

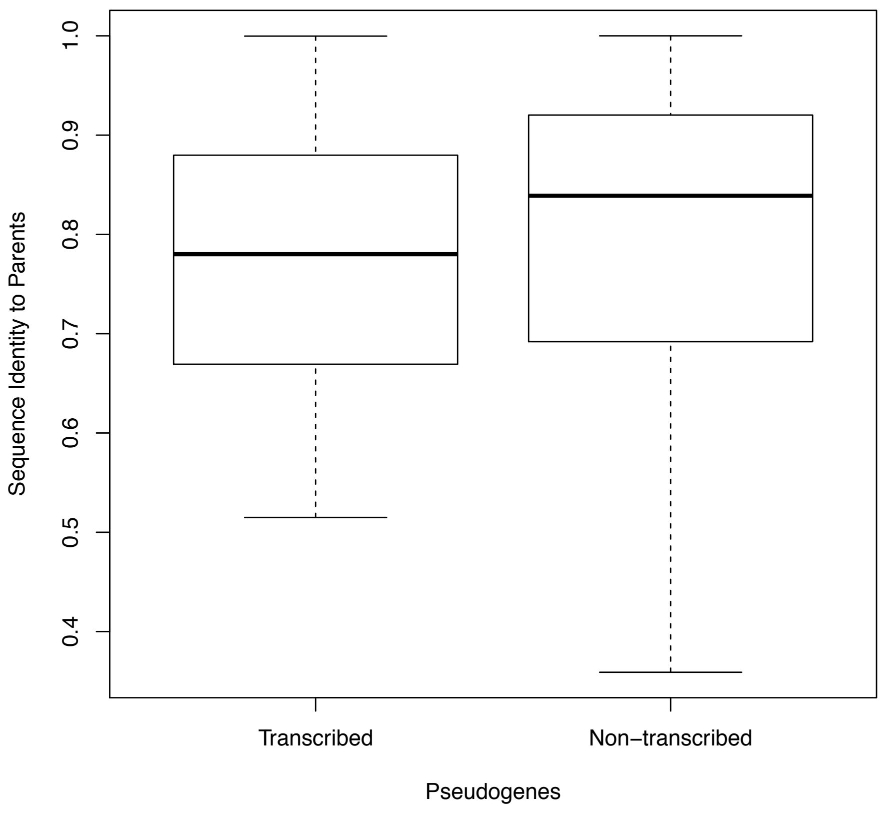

Figure S4: Sequence identity to parents. Transcribed pseudogenes on average show a lower sequence identity to parents than non-transcribed pseudogenes.

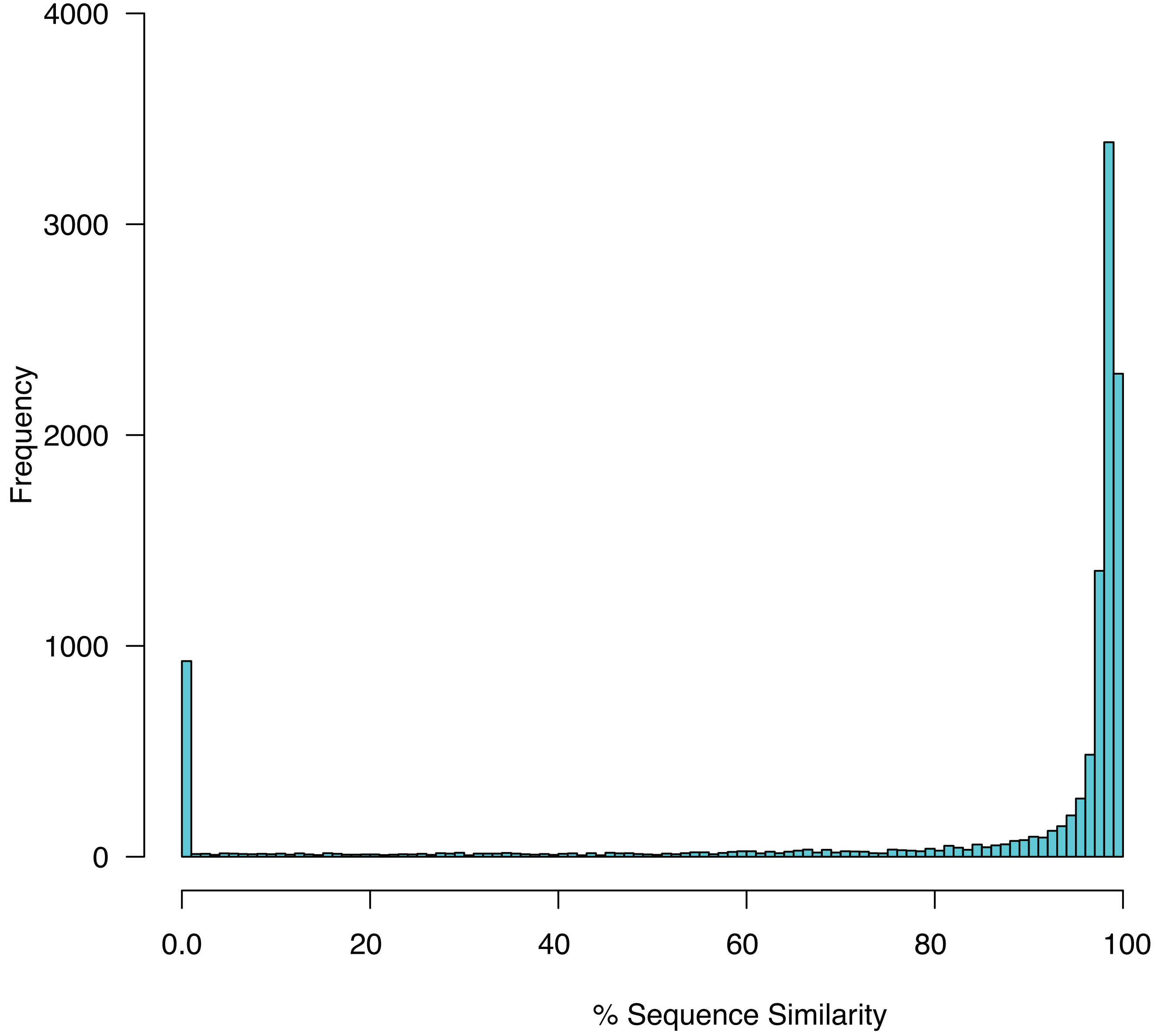

Figure S5: Sequence similarity for pseudogenes in human and chimp.

FIGURE S6: Variant densities in transcribed and non-transcribed pseudogenes. Densities of SNP, indel and SV in transcribed and non-transcribed pseudogene sequences are compared. Means and standard errors of densities are indicated. The transcribed pseudogenes have significantly lower SNP, indel and SV densities than non-transcribed pseudogenes with p-values are <2.2x10-16, 1.59x10-8 and <2.2x10-16, respectively. However, no significant differences were found in the DAF spectra (Fig. 7). To obtain better statistical power, we have repeated the analyses using a draft version of 1000 Genomes Phase I data which is derived from ~1000 individuals and much larger than the pilot data (1000genomes.org). We found similar results in DAF spectra for the two groups of pseudogenes.

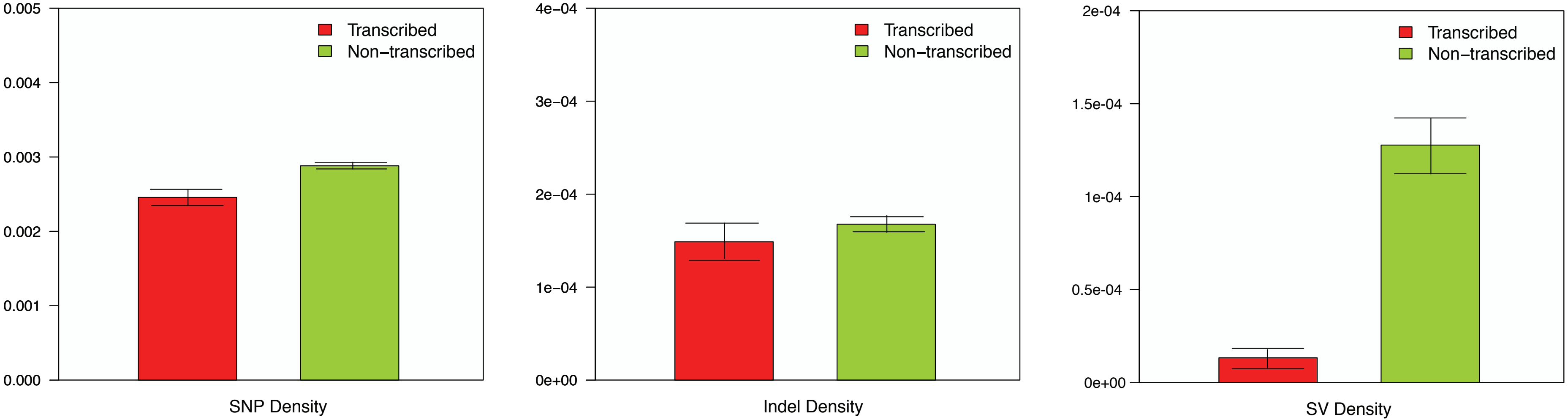

Figure S7: TFBS in upstream of pseudogene. Distribution of pseudogenes with different numbers of TFBS in their upstream sequences. Profiles from transcribed pseudogenes and non-transcribed pseudogene were compared for different cell lines.

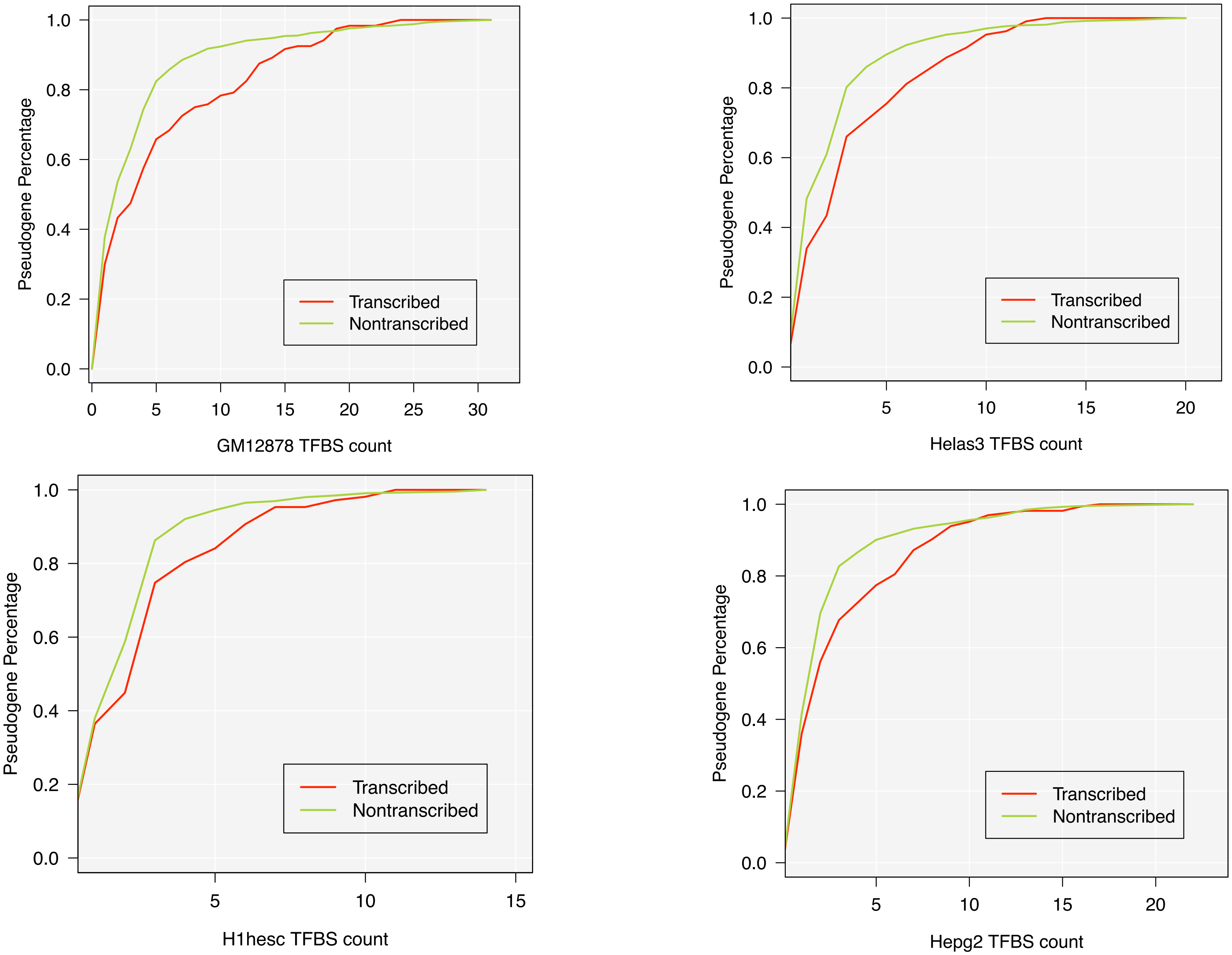

Table S0: Simple matrix to describe gene annotation of loci derived via retrotransposition. The presence/absence of disabling mutations is reflected on one axis and the presence/absence of locus-specific on the other. Combinations that would be annotated as processed_pseudogene are highlighted in green, transcribed_processed_pseudogene in orange and protein-coding loci in yellow.

	Disabling mutation	No disabling mutation
Locus-specific Transcription		
No Locus-specific Transcription		

Annotation	Colour
Processed_pseudogene	
Transcribed_processed_pseudogene	
Protein-coding gene (Retrogene)	

Table S1: Segway segmentation labels.

Segway Label	Description	Type
GE2	Gene End	Active Marks
GE1		
GE0		
GM1	Gene Middle	
GM0		
e/GM	Enhancer/Gene Middle	
GS	Gene Start	
TSS	Transcription Start Site	
TF2	Transcription Factor	
TF1		
TF0		
H3K9me1	H3K9me1	
C1	CTCF	Repressive Marks
C0		
R5	Repressed	
R4		
R3		
R2		
R1		
R0		
F1	FAIRE signal	
F0		
L1	Low signal	
L0		
D	Dead	

Table S2: Transcription factors enriched in the upstream regions of transcribed pseudogenes in different cell lines.

Cell line	Enriched TF*
Gm12878	Pol2, Elf1, Zeb1, Pou2f2, Sin3ak20, Gabp, Nrf1, Egr1, Tcf12, Znf, Sp1, Usf1, Pax5, Usf2, Yy1, Srf, Chd, Smc, Bcl3, Ctcf
K562	Pol2, Elf1, E2f6, Hey1, Max, Yy1, Gabp, Ccnt2, Tbp, Cmyc, Zbtb7, Taf1, Sin3ak20, Hdac2, Ets1, Hmgn3, Egr1, Nrf1, Irf1, E2f4, Sp2, Usf1, Rad21, Chd, Cfos, Ctcflsc98982, Sic5, Ctcf, Tf3c110, Nfyb, Thap1sc98174
Helas3	Pol2, Ini1, Gabp, Elk4, Nrf1, Taf1, Smc3, Tbp, E2f1, Mxi1, Cebpb, Usf2
Hepg2	Hey1, Pol2, Sin3ak20, Elf1, Taf1, Usf1, Gabp, Cmyc, Sp1, Tbp, Hdac2, Hnf4a, Hnf4g, Ctcf, Rxra, Srf, Fosl2, Jund, Rad21, Nrf1
H1hesc	Pol2, Taf1, Tbf, Usf1, Yy1, Nrf1, Gabp, Usf2, Ctcf, Taf7, Six5, Jund, Ctcf, Sp1

* FDR = 0.01

Table S3: Partially spliced pseudogenes.

Pseudogene Id	Chromosome	Strand	Start	End	Parent gene	Parent transcript
ENST00000333131.4	22	-	22469236	22472374	FAM108A1	ENST00000250974.8
ENST00000411545.2	22	-	21022106	21025272	FAM108A1	ENST00000250974.8
ENST00000503096.1	1	-	214779018	214782183	FAM108A1	ENST00000250974.8
ENST00000457740.2	1	+	147618674	147621845	FAM108A1	ENST00000250974.8
ENST00000358206.4	1	+	146076838	146080009	FAM108A1	ENST00000250974.8
ENST00000458502.1	17	+	20744360	20747574	FAM108A1	ENST00000250974.8
ENST00000417397.1	22	-	35897908	35899633	TRMT11	ENST00000334379.5
ENST00000512203.1	X	+	104650318	104651710	KCTD9	ENST00000221200.4
